# The Effects of Combined Cognitive-Physical Interventions on Cognitive Functioning in Healthy Older Adults: A Systematic Review and Multilevel Meta-Analysis

**DOI:** 10.3389/fnhum.2022.838968

**Published:** 2022-03-24

**Authors:** Jennifer A. Rieker, José M. Reales, Mónica Muiños, Soledad Ballesteros

**Affiliations:** ^1^Studies on Aging and Neurodegenerative Diseases Research Group, Madrid, Spain; ^2^Universidad Nacional de Educación a Distancia (UNED), Madrid, Spain; ^3^Universidad Internacional de Valencia (VIU), Valencia, Spain

**Keywords:** aging, cognitive training, three-level meta-analysis, multidomain training, combined training, physical exercise

## Abstract

Research has shown that both physical exercise and cognitive training help to maintain cognition in older adults. The question is whether combined training might produce additive effects when the group comparisons are equated in terms of exercise intensity and modality. We conducted a systematic electronic search in MEDLINE, PsycInfo, and Cochrane Central Register of Controlled Trials (CENTRAL) databases to identify relevant studies published up to February 2021. Seven hundred and eighty-three effect sizes were obtained from 50 published intervention studies, involving 6,164 healthy older adults, and submitted to a three-level meta-analysis. Results showed that combined training produced a small advantage in comparison to single cognitive training on executive functions, whereas both types of training achieved similar effects on attention, memory, language, processing speed, and global cognition. Combined training achieved higher training gains in balance than single physical training, indicating a transfer from cognitive training to balance. Performing cognitive and physical exercise simultaneously, and interactive training (e.g., exergames, square stepping) produced the largest gains in executive functions, speed, and global cognition, as well as the largest improvements in physical functions. Aerobic training was associated with higher effects in attention and fitness, whereas non-aerobic training produced larger effects in global cognition and balance. For all cognitive and physical outcomes, training resulted more advantageous when performed in a social context, even though individual training obtained similar results in balance as group training.

**Systematic Review Registration:** www.crd.york.ac.uk/prospero/, identifier: CRD42020175632.

## Introduction

Highly developed nations are experiencing large increases in the proportion of elderly citizens, due mostly to reduced birth rates and the increased longevity of their inhabitants (Reuter-Lorenz and Park, 2014). Demographic estimations predict that the proportion of the population above 60 will reach 35% by 2050 (Eurostat, 2016). Furthermore, the old-age dependency ratio (people aged 65 and above relative to those aged 15–64) will increase from 29.6% in 2016 to 51.2% in 2070 (European Commission, 2018). As aging affects several key cognitive functions negatively, such as processing speed, working memory, long-term episodic memory, and executive control functions (Baltes and Lindenberger, 1997; Park et al., 2002; Rönnlund et al., 2007), there is considerable interest in finding effective ways to improve and/or maintain these cognitive functions that are central for performing daily living activities.

Several longitudinal and cross-sectional studies conducted during the last two decades have shown that cognitive training interventions (e.g., Ball et al., 2002; Willis et al., 2006; Basak et al., 2008; Anguera et al., 2013; Ballesteros et al., 2014, 2017; Toril et al., 2016), regular physical activity (e.g., Colcombe and Kramer, 2003; Guiney and Machado, 2012; Voelcker-Rehage and Niemann, 2013; Prakash et al., 2015; Muiños and Ballesteros, 2018), and exposure to novelty (Park et al., 2014) can promote and/or maintain cognitive functioning in late adulthood.

A large body of research shows the positive link between physical activity and cognition. For a detailed description of the brain mechanisms associated with physical activity and its effects on cognition (see Kraft, 2012; Ballesteros et al., 2015). These reviews support the view that the combination of physical activity in conjunction to cognitive training may generate synergistic beneficial effects that either one alone.

### Physical Training

Physical activity can be defined as any bodily movement produced by skeletal muscles that require energy expenditure. Both moderate- and vigorous-intensity physical activity improve health (World Health Organization, 2019). A large body of research also corroborates the benefits of physical activity on brain structures and functions (Voelcker-Rehage et al., 2010; Erickson et al., 2011; Ruscheweyh et al., 2011; Liu-Ambrose et al., 2012; Bherer et al., 2013), and as a protection against age-related cognitive decline in executive functions and memory (Colcombe and Kramer, 2003; Hötting and Rödder, 2013; Voelcker-Rehage and Niemann, 2013; Bamidis et al., 2014). Aerobic exercise has been specially related to improvements in cognition (e.g., Colcombe and Kramer, 2003; Hindin and Zelinski, 2012), but coordination training (Voelcker-Rehage et al., 2011), resistance training, Tai Chi (Pons Van Dijk et al., 2013; Muiños and Ballesteros, 2015), and dance (Kattenstroth et al., 2013; Zilidou et al., 2018; Esmail et al., 2019; for reviews see Netz, 2019; Muiños and Ballesteros, 2020a,b) produce positive effects on brain and cognition in older adults.

### Cognitive Training

Cognitive training refers to a structured intervention that includes tasks designed to improve or maintain the cognitive functions that decline most with age. In the last years, several meta-analyses (Powers et al., 2013; Kelly et al., 2014; Lampit et al., 2014; Toril et al., 2014; Wang et al., 2016; Chiu et al., 2017; Tetlow and Edwards, 2017; Vázquez et al., 2018; Gavelin et al., 2020) examined the effects of cognitive-based training in older adults. Overall, their results indicated that video games and other cognitive-based training programs lead to small to moderate improvements in several aspects of cognition. A systematic overview of systematic reviews (Gavelin et al., 2020) on 46 reviews found a small mean effect of cognitive training in healthy and cognitively impaired older adults. Furthermore, larger effect estimates were related to higher review quality, and the authors concluded that cognitive training seems to improve cognition, but that the scarcity of high-quality evidence and heterogeneity in reported findings do not allow to estimate the clinical value of the effects.

However, other reviews (Gates et al., 2019; Lintern and Boot, 2019) were less optimistic about the effects of cognitive training. If effective, it seems that the transfer effects to untrained cognitive functions are either weak (Simons et al., 2016; Souders et al., 2017) or null when controlling for placebo effects and publication bias (Sala et al., 2018). Furthermore, several of the mentioned meta-analyses on cognitive training included also studies in which the participants also performed physical exercise (e.g., Legault et al., 2011; Maillot et al., 2012; Barnes et al., 2013; Shatil, 2013), confounding the effect of pure cognitive training with a potentially additive effect of cognitive training combined with physical activity.

### Combined Physical and Cognitive Training

The concurrent or simultaneous performance of physical exercise and cognitively challenging activities is known as combined, multidomain, or dual task training. Research on dual task performance has a long tradition in investigating how increased attentional demands affect either cognitive or physical performance due to prioritization in resource allocation to one or the other domain. Thus, these paradigms assume that our information processing system is limited and that conflicts in resource allocation are solved via interference control (McIsaac et al., 2015). On the other hand, neuroscientific approaches do not assume that one activity is necessarily executed on behalf of the other, but that combining physical and cognitive training might result in a mutual enhancement of both activities (Hötting and Rödder, 2013).

Animal studies have shown that physical exercise and cognitive stimulation contribute differentially to neuroplasticity in the mice brain, and whereas physical exercise promotes neurogenesis, cognitive stimulation promotes the differentiation of these new cells (Kronenberg et al., 2006; Kempermann et al., 2010). In humans, numerous studies have shown the beneficial effect of physical training on cognitive and functional brain plasticity in older adults, especially in hippocampal areas (Erickson et al., 2009, 2011; Niemann et al., 2014), suggesting similar mechanisms of neurogenesis as in animal models. Regular exercise has also been related to higher brain-derived neurotrophic factor (BDNF), which is involved in neurogenesis, synaptogenesis, and dendritic branching (Ruscheweyh et al., 2011; Håkansson et al., 2017), resulting in increased learning-related plasticity (Hötting and Rödder, 2013; Cassilhas et al., 2016). The release of BDNF serum is higher when physical exercise precedes cognitive training than vice versa (Nilsson et al., 2020), suggesting that physical exercise may have a facilitating effect on cognitive interventions.

A crucial question is whether combined physical and cognitive interventions, as opposed to single cognitive training or single physical training, produce synergistic effects on cognition, i.e., a combined effect that is greater than the effect produced by its components separately (Lustig et al., 2009; Kraft, 2012; Hötting and Rödder, 2013; Bamidis et al., 2014; Ballesteros et al., 2015). A systematic review (Lauenroth et al., 2016) analyzed 20 intervention studies on cognitive and physical combined training. The authors concluded that simultaneous or successive physical exercise and cognitive training were more effective than physical or cognitive exercise interventions alone. However, the results should be treated with caution due to the methodological heterogeneity of the original studies. Another review (Law et al., 2014) included 8 randomized controlled studies (RCT), but only 3 involved cognitively healthy older adults. Despite the small number of studies, the results indicated that participants' cognition in the combined cognitive and physical training condition was better than that of controls.

### Meta-Analytic Evidence on Combined Interventions

Several meta-analyses were conducted on the effects of combined interventions on the cognitive functions of older adults. The meta-analysis conducted by Zhu et al. (2016) included 20 interventional controlled trials (*n* = 2,667 healthy older adults). The results showed that combined interventions were superior to controls with a small effect size (0.29 random-effects model, *p* = 0.001) and physical exercise alone (overall effect size 0.22, *p* < 0.01), but not to cognitive training.

The meta-analysis of Guo et al. (2020) included 21 RCT conducted with healthy participants and adults with mild cognitive impairment (MCI) (*n* = 1,665). Combined interventions and cognitive training alone produced larger effects in executive functions compared to controls (Standardized Mean Difference; SMD = 0.26, *p* < 0.01). Differences were found between the effects produced by combined training and cognitive training alone (SMD = 0.13, *p* > 0.05) or physical training alone (SMD = 0.13, *p* > 0.05).

A network meta-analytic study (Bruderer-Hofstetter et al., 2018) included 11 combined or multi-component RCT studies conducted with healthy older adults (*n* = 670). According to their results, multi-component interventions were more effective than physical exercise and cognitive training alone and improved specific aspects of physical capacity and/or cognitive function. Physical and cognitive training conducted simultaneously or separately in older adults with normal cognition were effective, but in older adults with mild cognitive impairment (MCI), training performed separately was more effective.

On the other hand, the meta-analysis by Gheysen et al. (2018) included 41 intervention studies, 30 of which were conducted with healthy older adults. The authors investigated whether the combination of physical and cognitive interventions led to greater improvement in different cognitive processes compared to physical or cognitive interventions alone, and/or passive and active control groups. Results indicated that combining physical and cognitive training tasks in the same protocol produced larger benefits. Compared to the control condition, combined interventions produced larger cognitive gains (*g* = 0.316; *p* < 0.001). Combined interventions also induced significantly larger gains in cognitive functioning than physical exercise alone (*g* = 0.16; *p* = 0.008). However, combined and cognitive training alone did not differ (*g* = 0.02; *p* = 0.836). Nonetheless, the authors concluded that physical activity programs for older adults produce greater benefits when they incorporate cognitive tasks, and recommended activities such as dance and Tai-Chi that combine physical activity and cognitive training (see Muiños and Ballesteros, 2020a,b).

Vaportzis et al. (2019) included 7 combined physical and cognitive interventions, 25 physical, and 9 cognitive intervention studies in their meta-analysis of real-world interventions with healthy older adults. Five out of the seven combined studies reported superior results in the combined intervention vs. active controls. However, the meta-analysis did not find any significant difference in cognitive outcomes between combined and cognitive interventions alone.

### Methodological Questions and Meta-Analytic Inconsistencies

The meta-analyses discussed in the previous section thus produced some conflicting results, especially in terms of effect sizes. The conflicting results might be due to several factors as the heterogeneity of the studies included in each meta-analysis. Moreover, as in the case of the meta-analyses on cognitive training, meta-analytic works on combined cognitive-physical training often merge non-equivalent training interventions. Different study parameters, such as the dosage and the type of physical exercise (e.g., aerobic exercise *vs*. balance training), might modulate the training outcomes differentially. Also, on a within-study level, combined training is often compared with a different type of physical exercise than the one performed in the combined condition. The inclusion of a control condition in the design reduces expectation bias that could inflate training outcomes and account for other threats to internal validity (Gold et al., 2017). However, in contrast to pharmacological interventions, in behavioral studies, it is extremely difficult to find psychological placebos or “sham” interventions, as any activity might have the potential to produce unexpected effects on cognition and behavior. For example, in some studies, the training effect produced by exergames was compared with that produced by balance (Eggenberger et al., 2015; Schättin et al., 2016) or strength training (Bacha et al., 2018). In other studies, aerobic training was compared with stretching plus strength (Barnes et al., 2013), or stretching, strength, and balance training (Ten Brinke et al., 2020). In other cases, both groups received a similar training part, such as aerobic and strength training, and another different one (Boa et al., 2018). Or both groups did not differ in the physical training type or load, but the single physical training group also received cognitively enhancing dual-task training (Kayama et al., 2014). Furthermore, activities used as a control condition in some studies, as balance and/or strength training, were used in other studies as experimental conditions (Hiyamizu et al., 2012; Gschwind et al., 2015; Jehu et al., 2017; Wongcharoen et al., 2017; Laatar et al., 2018), adding a further challenge for meta-analytic analyses. It seems logical to think that aerobic exercise exerts a different effect on body and cognition than, for example, balance or strength training. Hence, the comparison of two groups that receive different training regimes does not allow to isolate the combinatory effect of physical exercise and cognitive training when both groups perform different physical or cognitive activities. Nonetheless, all meta-analyses conducted to date included at least one of the studies mentioned above, computing effect sizes from the comparison of non-equivalent physical training components.

Meta-analyses might also suffer from analytical flaws. Most interventional studies include more than one outcome measure, which produces an interdependency of effect sizes. Traditional univariate approaches often apply the *sample wise* procedure, averaging the dependent effect sizes within studies into a single effect size by computing a weighted average (Cheung, 2019). However, this method underestimates the degree of heterogeneity or the variance of the population and might lead to lower statistical power due to information loss (Cheung, 2019). A relatively novel approach for dealing with the dependency of effect sizes consists in applying a three-level structure to a meta-analytic model (Assink and Wibbelink, 2016). This approach considers three different variance components and allows effect sizes to vary between participants (sampling variance), outcomes (within-sample variance), and studies (between-study variance). The three-level meta-analytic model allows analyzing the training effects on different cognitive functions within the same study (i.e., within-study heterogeneity) and their reliability across different studies (i.e., between-study heterogeneity).

### Aims and Hypotheses of This Multilevel Meta-Analysis

The primary aim of this systematic review and three-level meta-analysis was to shed light on whether combined physical and cognitive training is more effective than single-domain training (physical or cognitive alone) in maintaining and/or improving cognition in healthy older adults while controlling for the dependency of effect sizes, and differences in the training protocols. Specifically, the present multilevel meta-analysis addressed the following research questions:

1. Does combined training produce synergistic or additive effects, i.e., are the effects obtained by the combination of cognitive and physical training larger than those obtained by each of its components separately?
2. Are the effects of cognitive training differentially modulated when combined with aerobic vs. non-aerobic exercise?
3. Does simultaneous cognitive and physical training produce better results than sequential training performed on the same day (sequential training schedule) or different days of the week (separate training schedule)?
4. Does the type of cognitive training (computer, interactive, such as exergames, or multicomponent training) influence the training outcomes?
5. Does training produce better results when performed in groups than when performed individually?
6. Finally, to what extent are the results influenced by the quality of the studies, publication bias, year of publication, sample size, age, or training duration?

## Methods

The review was registered in the International Prospective Register of Systematic Reviews (PROSPERO, https://www.crd.york.ac.uk/prospero/; CRD42020175632). To conduct this systematic review and multilevel meta-analysis, we followed the Preferred Reporting Items for Systematic Reviews and Meta-Analysis (PRISMA; www.prisma-statement.org) guidelines for reporting studies (Moher et al., 2009). The objective was to ensure comprehensive and transparent reporting methods and results. The process and methods were established before conducting the review.

### Literature Search Strategy

A systematic electronic database search was conducted to identify relevant published studies. The MEDLINE, PsycInfo, and Cochrane Central Register of Controlled Trials (CENTRAL) databases were searched to identify relevant studies published up to February 2021, with no period specified for the date of publications.

The search terms were intersections of terms referring to the combination of cognitive and physical activities in older adults intended to improve cognitive and physical health. The search terms were intersections of terms referring to the combination (*combined OR combination OR simultaneous OR dual OR concurrent OR sequential OR multimodal OR multidomain OR multicomponent*) of cognitive *(cognitive OR mental OR memory OR “executive functions” OR “video games”)* and physical (physical OR exercise OR motor OR mobility OR strength OR aerobic OR endurance OR cardiovascular OR kinetic OR kinect OR exergame^*^) interventional studies *(training OR program OR intervention OR fitness OR activity)* conducted with older adults *(older OR elderly OR elderlies OR aging or aging OR aged OR seniors)*. For the full search strategy (see Supplementary Material S1).

Next, the electronic search was complemented by reviewing the reference lists of the retrieved articles and reviews and then hand-searching cited articles considered to be of interest. Titles and abstracts were first screened by two of the authors (JAR and MM), who then individually screened the full text of relevant articles. In the event of disagreement, a consensus was achieved following a discussion with JMR and SB. If the study was relevant for our analysis but the data necessary to calculate the effect sizes were missing, the authors were contacted via email to obtain the relevant data. Of the four datasets requested, two were provided by the authors. The two remaining datasets were not provided by the authors, so we resorted to extracting the data from the graphs provided in the papers using the online tool WebPlotDigitizer version 4.3.

### Selection Criteria

We restricted inclusion in this review to research articles written in English and published in peer-reviewed journals. They also had to meet the following criteria:

- **Study participants**: Healthy older adults (mean age 60 years or older) with no known cognitive impairment or other mental illness or neurological disorder including depression, stroke, dementia, or Parkinson's disease. Studies involving both healthy and cognitively impaired older adults (with mild cognitive impairment or dementia) were only included if the results for the healthy sample were reported separately. In that case, we only used data from the healthy sample.
- **Combined interventions**: The studies included at least one combined physical and cognitive training group.
- **Comparison groups**: Studies were considered when they included, in addition to the combined training group, at least one of the following: (a) a single-physical exercise group; (b) a single-cognitive training group; (c) a passive control group (e.g., waiting list, business as usual); (d) an active control group (alternative interventions, such as leisure activities, health education or toning exercises).
- **Equivalent training components**: when the comparison groups consisted of single physical and/or single-cognitive training, only those studies in which the training components of the combined and the single-component training were identical (i.e., the same dosage of aerobic exercise, strength, or balance training) were included.
- **Study design:** We included only intervention studies with pre/post assessments of cognitive outcomes, excluding single-session trials (e.g., studies with only a post-test assessment). The studies could be randomized controlled trials (RCT), cluster-RCT, or non-RCT.
- **Descriptive statistics**: Studies were included if they provided the statistics needed to compute the *g* effect size index and its confidence interval or provided sufficient information to calculate at least one effect size for at least one cognitive outcome measure.
- The outcome measures assessed cognitive or physical functions objectively, as described in more detail below.

### Data Extraction

#### Outcome Measures

The cognitive outcomes included objectively assessed cognitive domains of processing speed, attention, memory, executive control, verbal abilities, global cognition, as well as composite scores from test batteries. Processing speed included tests that measured reaction times. Attention included divided, selective, and sustained attention measures. The classification of executive functions assessments was based on published factor analyses (e.g., Miyake et al., 2000; Friedman and Miyake, 2004) and included tests that measured working memory, inhibition, and flexibility. Memory included short- and long-term memory tests. Language included assessments of verbal, categorical, and phonological fluency. Global cognition comprised the results of cognitive screening tools, and lastly, composite scores included z-scores from test batteries.

Objectively assessed physical measures were classified into fitness, strength, and balance. In the case of dual-task paradigms (the simultaneous performance of a physical and a cognitive task), we only computed the scores of the cognitive task, but not the physical scores. Given the close relationship between balance and gait, we coded gait parameters within the balance category, such as stride variability or step length. Results of simple motor reaction time tests were not included.

When authors provided the results of subcategories of screening tools (e.g., MMSE), we only coded the global score within the category “global cognition.” Several studies included combined interventions with and without other treatments. In this case, we only computed the combined training group that did not receive other treatments. When a study included additional training groups whose training components differed from those of the combined group, we only computed the data from equivalent groups. When a test was tailor-made or unusual, we analyzed the task paradigm in detail by examining the procedures, item-specific analyses, and online and graphic material. For a detailed description of the tests used in each study (see Supplemental Material S2).

#### Moderators

(a) mode of delivering the combined training (simultaneous, sequential, and separate). Simultaneous training included interactive interventions, such as exergaming (e.g., pedaling and steering a bicycle in a virtual world and attainment of goals), body-mind activities in psychomotor modality, in which the cognitive training is performed while carrying out physical movements, and dual-task interventions, in which cognitive and physical components are typically separate tasks but performed at the same time. Combined interventions in sequential mode included cognitive and physical exercises performed one after the other in the same session. For combined interventions in the separate mode, the two training components were delivered on different days of the week. In square stepping exercise (SSE), the cognitive demands depend on the difficulty of the foot placement patterns being performed and progression through the stepping protocols. At beginner levels, as in Gill et al. (2016), the activity can be conceptualized as a lower extremity coordination exercise and we considered it a physical component. In SSE with increasingly more complex stepping patterns, as in Schoene et al. (2015), the activity can be conceptualized as a visuospatial working memory task requiring a stepping response and considered a simultaneous cognitive-physical intervention; (b) Aerobic *vs*. non-aerobic exercise. The aerobic intensity was classified according to the information provided by the authors. Low aerobic exercises such as walking or light group activities (e.g., catching balls) were classified as non-aerobic. Other moderators were: (c) number of training sessions; (d) intervention length in weeks; (e) minutes of training per week; (f) study quality; (g) mean age and its standard deviation (SD), and (h) year of publication. A couple of studies did not report the precise number, duration, and/or frequency of training sessions, but only minimum and maximum values; in these cases, we coded the mean value of each group.

### Assessment of Methodological Quality

Two authors (SB and MM) independently conducted a qualitative assessment of the methodological quality of the studies included in this review using the Standard Quality Assessment Checklist (Kmet et al., 2004). In this checklist tool, the maximum score for study quality is 28. Methodological quality is considered excellent if the score is >80%, good if it is 70–79%, fair if it is 50–69%, and poor if it is <50%. When there was a disagreement in scoring a study, the authors discussed the matter until they reached an agreement. For a detailed description of the quality assessment of the reviewed articles (see Supplemental Material S3).

### Interrater Reliability

The studies were coded by two independent reviewers (JAR and JMR). Disagreements were solved by discussion. When this process was finished, a third reviewer (MM) randomly selected and coded ten studies from the whole set, and interrater reliability for this subset of studies was calculated. Cohen's Kappa for the categorical variables and intraclass correlations for continuous variables ranged from 0.94 (classification of measured functions) to 1 (research design).

### Effect Sizes

To quantify the differential training effect of combined vs. cognitive and/or physical training alone, and/or active/passive control on cognitive and physical outcome measures, we computed the standardized mean differences of effect sizes and their variance for each physical and cognitive outcome of the original papers using the formula


$$\begin{array}{l} g=\left[c_{m}\right]\left[\frac{\left({\bar{y}}_{Post}^{Exp.}-{\bar{y}}_{Pre}^{Exp.}\right)-\left({\bar{y}}_{Post}^{Cont.}-{\bar{y}}_{Pre}^{Cont.}\right)}{S_{pooled}}\right]\\ S_{pooled\;}=\;\sqrt{\frac{\left(n_{Exp.}-1\right){\left(S_{Pre}^{Exp.}\right)}^{2}+\left(n_{Cont.}-1\right){\left(S_{Pre}^{Cont.}\right)}^{2}}{n_{Exp.}+n_{Cont.}-2}}\\ c_{m}=\left[1-\frac{3}{4\left(n_{Exp.}+n_{n_{Cont.}.}\right)-9}\right] \end{array}$$

where ${\bar{y}}_{Post}^{Exp.}$ and ${\bar{y}}_{Pre}^{Exp.}$ are the experimental group posttest and pretest means, ${\left(S_{Pre}^{Exp.}\right)}^{2}$ is the variance of the pretest scores, *c*_*m*_ is a bias correction factor inversely proportional to the sample size, *n*_*Exp*._ is the sample size of the experimental group, and ${\bar{y}}_{Post}^{Cont.}$, ${\bar{y}}_{Pre}^{Cont.}$, ${\left(S_{Pre}^{Cont.}\right)}^{2}$, *n*_*Cont*._ are the corresponding values for the comparison group. As we used a bias correction factor, the Standardized Mean Difference (*SMD)* computed was thus Hedge's *g* instead of Cohen's *d*. The standard deviation of Hedge's *g* was computed with the following equation:


$$\begin{array}{l} S_{g}=\sqrt{c_{m}^{2}\left(\frac{n_{Exp.}+n_{Cont.}}{n_{Exp.}•\;n_{Cont.}}\right)\left(\frac{n_{Exp.}+n_{Cont.}-2}{n_{Exp.}+n_{Cont.}-4}\right)\left(1+\frac{\left(n_{Exp.}•n_{Cont.}\right)g^{2}}{n_{Exp.}+n_{Cont.}}\right)-g^{2}} \end{array}$$

Each study usually included several dependent variables for the same outcome, either because the experiment produced several dependent variables for the same task (e.g., reaction times (RT), error rates, delayed and immediate recall, etc.), or because different assessment tools were used to evaluate the same function. We computed at least two effect sizes (ES) for each dependent variable reported in the original articles: one for the effect of the combined cognitive-physical treatment, and one for the single-cognitive and/or the single-physical and/or the active and/or passive control group. In all cases, the means and sample sizes for the combined group were the same, and only the means and sample sizes for the three possible comparison groups (cognitive, physical, and control) differed. This indicates that these ES had dependence between them stemming from two sources: several ES were computed from the same original study (for different dependent variables), and they used a common group (the combined group) as a reference point to compute ES.

### Statistical Analyses

Modeling ES using a three-level structure is a better approach than a two-level structure when there are several dependent effect sizes in each independent study, but only if the heterogeneity of the sampling variance is substantial. In three-level meta-analytic models, three different sources of variance are modeled: the third level describes the variance of effect sizes between studies (between-study), the second level describes the variance of effect sizes of the experiments, or measurements nested within each study (within-study), and the first level describes the sample variance. We performed the multilevel random-effects analysis with and without moderators using restricted maximum likelihood estimation. This analytical solution was specifically designed to account for the non-independence among ES, and it was the preferred methodology as the sampling variability was not too high.

Heterogeneity among our effect sizes was assessed using the Q statistic. A large Q-value indicates that differences between ES do not derive from a common population mean from the original study samples but are accounted for by other reasons. The Q statistic is distributed as a χ^2^ distribution.

Statistical analysis was performed using the rma.mv function of the metaphor package (version 2.4) (Viechtbauer, 2010) within the R software environment (version 4.0.1; R Core Team, 2021). We followed the analytical steps presented by Assink and Wibbelink (2016). Dot-plot figures were depicted using Mathematica (version 10.4) with software developed specifically for this study.

### Outlier Analysis

Outliers or influential cases are considered cases that could distort the results in one or another direction. We performed outlier and influential case diagnostics using the *influence* function of the metaphor package. This function calculates the influence of deleting one case at a time on the model fit or the fitted/residual values, based on several indices: the externally standardized residual, DFFITS value, Cook's distance, covariance ratio, the leave-one-out amount of (residual) heterogeneity, the leave-one-out test statistic of the test for (residual) heterogeneity, and DFBETAS value(s). In one study, the identified influencer cases constituted the only cognitive effect sizes (Norouzi et al., 2019). Regarding the follow-up outcomes, the influence function suggested deleting all cases belonging to one specific study. Given that according to the metafor package description, the chosen cut-offs are (somewhat) arbitrary, and that substantively informed judgment should always be used when examining the influence of each case on the results, we decided not to use this function for the follow-up cases but base our decisions on the visual inspection of funnel plots. Supplementary Table S4 summarizes the cases that were detected and removed from the database before the meta-analysis.

### Publication Bias

Despite our comprehensive review and systematic search strategy, it is possible that some studies were missed due to publication bias. Generally, studies that fail to produce significant results are either not submitted for publication by the authors or rejected by the editors or reviewers. This could lead to bias toward the publication of significant statistical effects, something known as the “file-drawer problem.” Although there are many ways to estimate publication bias (Rothstein et al., 2006), most do not apply to multilevel studies due to dependent effect sizes. We addressed this issue with several procedures. First, we visually inspected the funnel plots of cognitive and physical functions. In the funnel plots, effect sizes were charted against the standard error around the estimated summary effect of cognitive and physical ES. An asymmetric funnel plot (e.g., usually an under-representation of non-significant and/or negative effects on the bottom left side of the plot) would suggest the existence of publication bias. To test the statistical significance of the plots, we applied Egger's test (Egger et al., 1997), which analyzes whether the standardized effect sizes can predict study precision (defined as the inverse of the standard error) in a linear regression. Furthermore, we generated fail-safe numbers (i.e., the number of non-significant ES needed to change a significant into a non-significant result) following different approaches (Rosenthal, 1979; Orwin, 1983; Rosenberg, 2005). Finally, we used the trim-and-fill method of Duval and Tweedie (2000a,b) to determine how many ES would need to be imputed to restore the symmetry of the funnel plot.

## Results

### Search Results

**Figure 1:**
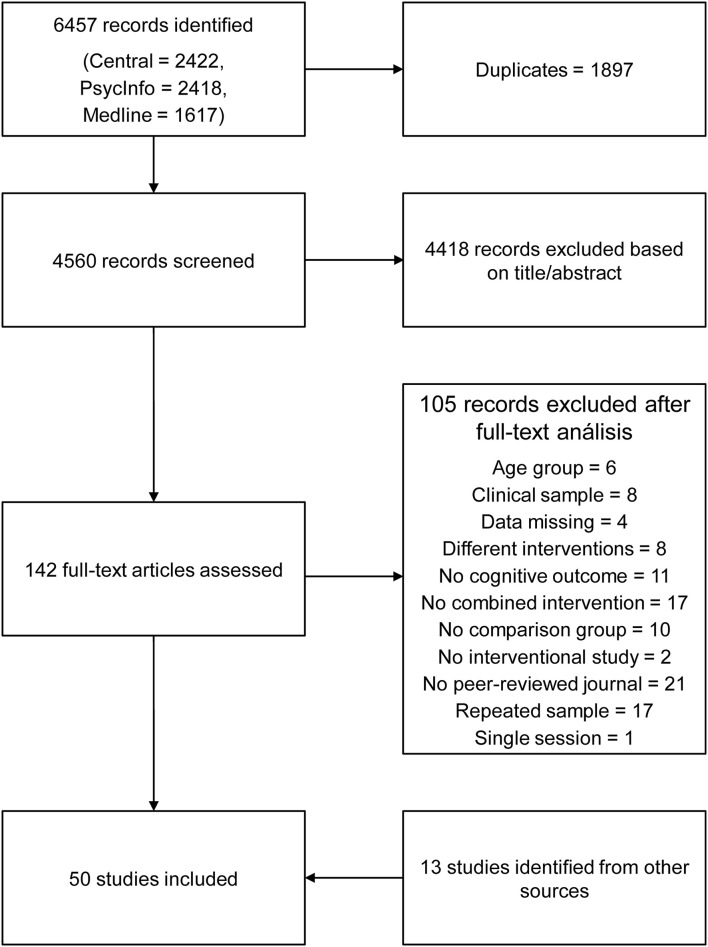
PRISMA flow diagram of the search strategy.

The initial search yielded 6,457 studies. After excluding duplicates and studies that did not meet the inclusion criteria, 50 studies were included in the analysis. Figure 1 shows the PRISMA flow diagram of the systematic search and study selection.

### Descriptive Results: Studies and Participant Characteristics

In most studies, there was more than one outcome measure. After removing 26 outliers (3.21%), our meta-analysis included a total of 783 effect sizes, of which 697 corresponded to pre-post assessments and 86 to pre/follow-up assessments. Table 1 shows the descriptive data of all the primary studies included in our analysis. The eligible studies were published up to February 2021. The largest number of published studies was in 2015 with 10 studies, followed by 2017, 2020, and 2014 (7, 6, and 5 published studies, respectively). Four studies were published in 2012 and 2018, three in 2012 and 2021, and two in 2009 and 2016. In 2002, 2006, 2011, and 2019 there was just one published study per year. The countries with the largest number of published studies were Japan and USA with six studies each, followed by Germany with five studies, and Switzerland and France with four studies each, Australia and Canada with three studies each, Brazil and Thailand with two studies, and China, Finland, Greece, Iran, Italy, Mexico, Myanmar, Portugal, Singapore, South Korea, Spain, and Tunisia with one study each. Two studies were multisite, participating Italy, Greece, Spain, and Serbia in one, and Spain, Germany, and Australia in the other study. A total of 6,164 healthy older adults participated in the 50 studies with a mean age of 72.12 (*SD* = 4.51) years. Bamidis et al. (2015) did not report the mean age, but their participants were older than 55 years, so the mean age was computed over 50 studies. The number of participants in each study ranged from 13 (You et al., 2009) in a pilot study to 1.190 (Ngandu et al., 2015) with a global mean of 123.928 (*SD* = 201.885). Of all studies, six studies included a follow-up assessment. However, as the total of follow-up outcomes only summed up 86 effect sizes, these were only analyzed in a summary fashion and not by cognitive or physical functions. Twenty-seven studies reported a comparison of combined training *vs*. active or passive control (*n* = 4,555), nine studies compared combined training with single cognitive training (*n* = 441), and 14 studies compared combined training with single physical training (n = 1,168). Two studies included two types of combined training compared with a control group (Wollesen et al., 2017) and single cognitive training (Yu et al., 2021). The combinatory mode for the combined groups was sequential (13 studies, *n* = 1,780), separate (9 studies, *n* = 2,760), or simultaneous (28 studies, *n* = 1,624). The total duration of the intervention ranged from 4 weeks (Wongcharoen et al., 2017; Norouzi et al., 2019) to 144 weeks (Andrieu et al., 2017) with a global mean of the duration of 20.29 weeks (*SD* = 26.04). The total number of training sessions ranged from 8 (Kitazawa et al., 2015) to 745 (Andrieu et al., 2017), with a global mean of 61.9 sessions (*SD* = 124.05). The duration (in minutes) of cognitive intervention sessions ranged from 30 (Schoene et al., 2013; Linde and Alfermann, 2014; van Het Reve and de Bruin, 2014) to 360 (Pieramico et al., 2012; Shah et al., 2014), with a global mean of 114.8 min per week (*SD* = 64.46). The duration of physical intervention sessions ranged from 40 min (Schoene et al., 2013) to 250 (Shah et al., 2014) with a mean duration of 118.31 min (*SD* = 49.40). The studies varied in the type of physical training, and in 38 studies, the training included fitness, and/or balance, and/or strength. The aerobic exercise intensity was moderate to high in 17 studies (*n* = 1,235) and low to none in 29 (*n* = 3,176) studies. In four studies, it was not possible to determine the aerobic exercise intensity. Cognitive training included a variety of exercises (memory, planning, reasoning, visuospatial skills, attention, switching tasks, arithmetic, verbal fluency, problem-solving, and other cognitive tasks). In 15 studies (*n* = 650) the cognitive training was performed interactively (exergames, psychomotor exercises, and square stepping), in 17 studies (*n* = 3,197) via computer games or computer tasks, and in 18 studies (*n* = 2,317) *via* a multicomponent training (paper-pencil tasks, group games, verbal games, etc.) or verbal exercises.

**Table 1:** Study designs and descriptive data of the primary studies included in the meta-analysis.

**References**	**Country**	***N***	**Groups (n, M_**age**_)**	**No. of sessions**	**Duration (wks)**	**Follow-up (wks)**	**Cognitive intervention**			**Physical intervention**				**Combinatory mode**	**Setting**	**Control activities/other components**	**Outcome measures**
							**Description**	**min/wk**	**Trained functions**	**Description**	**min/wk**	**Trained functions**	**Aerobic intensity**				
Adcock et al. (2020)	Switzerland	31	EI-CI (15, 77) PC (16, 70.9)	48	16	-	Square stepping, 3 d/wk	95	EF, attention	Tai Chi-inspired movements and dancing 3 d/wk	105	Strength, balance, fitness	Low	Simultaneous	Individual	-	**Cognitive**: EF, PS, memory **Physical**: balance (gait), fitness
Anderson-Hanley et al. (2012)	USA	63	EI-CI (30, 76.1) EI (33, 81.7)	36	12	-	Exergames 3 d/wk, 2 months	135	Not clear	Stationary bicycle riding at 60% HRmax 3 d/wk for 3 months	135	Fitness	Moderate	Simultaneous	Individual	-	**Cognitive**: EF, global cognition, attention, language, memory
Andrieu et al. (2017)	France	722	EI-CI (356, 75) PC (366, 75.1)	745	144	-	Multicomponent exercises 1.5 d/wk during the first 2 months, 1 d/every 3rd mo. for the rest of the trial.	90	ES, PS, memory	Personalized home-based exercise program 5 d/wk	150	Fitness, balance, strength	Low	Separate	Mixed	-	**Cognitive:** global cognition, memory, PS language, attention, EF **Physical**: fitness
Bamidis et al. (2015)	Greece	90	EI-CI (69, n/a) PC (21, n/a)	37 CI: 14, EI:23	9	-	Computerized cognitive training (Posit Science), 3 d/wk	180	EF, memory	Exergames (FitForAll for Wii) at 55–85% HRmax, 2.3 d/wk	120	Fitness, balance, strength	Moderate	Not clear	Group	-	**Cognitive**: composite score of EF and memory
Barban et al. (2017)	Italy, Greece, Spain, Serbia	481	EI-CI (121, 74.5) EI (119,75.5) CI (118, 74.1) CC (123, 76)	24	12	12	Computerized cognitive training 2 d/wk	EI-CI:60 CI:120	EF, memory	Supervised structured exercise program with i-walker. 2 d/wk	EI-CI:60 EI:120	Balance, fitness	Low	Sequential	Mixed	CC: entering data into computer	**Cognitive**: memory
Desjardins-Crépeau et al. (2016)	Canada	76	EI-CI (22, 72.7) EI-CC (16, 70.9) EC-CI (20, 73.2) EC-CC(18, 72.5)	36	12	-	Computer tasks 1 d/wk	60	EF, attention	Supervised structured exercise program and treadmill walking 2 d/wk	120	Fitness, strength	Moderate	Sequential	Group	EC: Stretching, toning CC: Computer lessons	**Cognitive**: ES, memory, PS **Physical**: fitness, balance, strength
Eggenberger et al. (2015)	Switzerland	47	EI-CI (22, 78.5) EI (25, 80.8) DANCE not incl.	52	26	24	Computer tasks 2 d/wk	120	Memory	Structured exercise program and treadmill walking 2 d/wk	120	Fitness, strength, balance	Moderate	Simultaneous	Mixed	-	**Cognitive**: memory, attention, EF, PS
Fabre et al. (2002)	France	32	EI-CI (8, 64.9) CI (8, 67.5) EI (8, 65.4) AC (8, 65.7)	24	8	-	Multicomponent exercises 1 d/wk	90	Memory, attention, language	Supervised outdoor interval training at ventilatory threshold 2 d/wk	120	Fitness	Moderate	Separate	Group	AC: leisure activities	**Cognitive**: memory **Physical**: fitness
Gill et al. (2016)	Canada	44	EI-CI (23, 72.6) EI (21, 74.5)	78	26	-	Verbal exercises 3 d/wk	45	EF, language	Structured aerobic exercise at 70–85% HRmax and beginner-level square stepping 3 d/wk	120	Fitness	Moderate	Simultaneous	Mixed	-	**Cognitive**: EF, PS, memory, language
Gschwind et al. (2015)	Spain, Germany, Australia	153	EI-CI (78, 74.7) PC (75, 74.7)	42	16	-	Computerized exercises 2.5 d/wk	100	EF, attention	Individualized training protocol embedded in home-based exergames 2.5 d/wk	112	Strength, balance	None	Simultaneous	Individual	-	**Cognitive**: EF, PS, attention. **Physical**: balance, fitness, strength
Hiyamizu et al. (2012)	Japan	36	EI-CI (17, 72.9) EI (19, 71.2)	24	12	-	Verbal exercises 2 d/wk	120	EF, attention, language	Supervised structured exercise program 2 d/wk	120	Strength, balance	None	Simultaneous	Group	-	**Cognitive**: EF, PS **Physical**: balance, strength
Htut et al. (2018)	Myanmar	42	EI-CI (21, 75.8) PC (21, 76)	24	8	-	Exergames 3 d/wk	90	PS, attention	Exergames 3 d/wk	90	Balance, fitness	Low	Simultaneous	Individual	**-**	**Cognitive**: global cognition **Physical**: balance, strength
Jardim et al. (2021)	Brazil	72	EI-CI (41, 67.4) PC (31, 67.9) EI + CI not incl.	24	12	-	Verbal exercises, psychomotor tasks 2 d/wk	150	EF, memory, attention, language	Supervised structured exercise program at 60–70% HRmax 2 d/wk	150	Fitness, balance, strength	Moderate	Simultaneous	Group	-	**Cognitive:** memory, attention **Physical:** fitness, balance, strength
Jehu et al. (2017)	Canada	41	EI-CI (14, 68.7) EI (15, 70.2) PC (12, 66.3)	36	12	12	Verbal exercises 3 d/wk	180	EF, language	Supervised structured exercise program 3 d/wk	180	Balance	None	Simultaneous	Individual	-	**Cognitive**: EF **Physical**: balance
Joubert and Chainay (2019)	France	48	EI-CI (16, 69.4) PC (16, 69.8) CI (16, 69.5)	16	8	4	Home-based computerized cognitive training (HAPPY neuron Professional) EI-CI: 1 d/wk CI: 2 d/wk	EI-CI:60 CI:120	EF	Supervised treadmill walking 1 d/wk	60	Fitness	Moderate	Separate	Individual	-	**Cognitive**: EF, language
Kitazawa et al. (2015)	Japan	60	EI-CI (30, 76.8) PC (30, 75.5)	8	8	-	Square stepping 1 d/wk	60	Memory	Supervised square stepping 1 d/wk	60	Fitness, balance	Low	Simultaneous	Group	-	**Cognitive**: global cognition, memory **Physical**: balance
Laatar et al. (2018)	Tunisia	24	EI-CI (12, 66.3) EI (12, 67, 45)	72	24	12	Verbal exercises, psychomotor tasks 3 d/wk	180	EF, memory, attention	Supervised structured exercise program 3 d/wk	180	Strength, balance	None	Simultaneous	Group	-	**Cognitive**: PS **Physical**: fitness, balance (gait), strength
Legault et al. (2011)	USA	67	EI-CI (18, 75.4) CI (16, 76.0) EI (16, 77.5) AC (17, 76.9)	56	16	-	Center-based computer tasks 1.5 d/wk	100	Memory	Center-based and home-based exercises including walking or stationary cycling 2 d/wk	150	Fitness	Moderate	Separate	Mixed	AC: Health education	**Cognitive**: EF, memory
Linde and Alfermann (2014)	Germany	55	EI-CI (16, 65.6) EI (15, 68.3) CI (11, 67.3) PC (13, 66.6)	32	16	12	Multicomponent exercises 1 d/wk	30	EF, PS, memory, attention	Supervised structured exercise program at 40% to 70% HRmax. 2 d/wk	120	Fitness, strength	Moderate	Sequential	Group	-	**Cognitive**: EF, memory, attention, PS **Physical**: fitness
Maillot et al. (2012)	France	30	EI-CI (15, 73.5) PC (15, 73.5)	24	12	-	Exergames 2 d/wk	120	Not clear	Wii exergames 2 d/wk	120	Fitness, balance	Not clear	Simultaneous	Not clear	-	**Cognitive**: EF, PS **Physical**: fitness, strength
Marmeleira et al. (2009)	Portugal	32	EI-CI (16, 68.4) PC (16, 68.2)	36	12	-	Psychomotor tasks 3 d/wk	180	EF, PS, attention	Psychomotor responses to cognitive demands (walking, catching balls, etc.) 3 d/wk	180	Fitness	Low	Simultaneous	Group	-	**Cognitive**: attention, EF, PS **Physical**: fitness, balance
McDaniel et al. (2014)	USA	79	EI-CI (19, 6) EI-CC (23, 7) CI-EC (18, 6) CC-EC(19, 6)	96	24 (CI: 2 EI: 6)	-	Multicomponent exercises 3 d/wk	180	EF, memory, attention	Supervised treadmill walking or stationary cycling at 50% to 85% HRmax. 3 d/wk	180	Fitness	Moderate	Sequential	Group	EC: Flexibility CC: Health education	**Cognitive**: attention, memory **Physical**: VO_2_peak
Morita et al. (2018)	Japan	19	EI-CI (8, 75) PC (11, 71.9)	96	96	-	Verbal exercises, psychomotor tasks 1 d/wk	60	EF, memory, language	Supervised structured exercise program 1 d/wk	60	Fitness, strength	Low	Simultaneous	Group	-	**Cognitive**: global cognition **Physical**: strength, fitness, balance
Ng et al. (2018)	Singapore	197	EI-CI (49, 70.4) EI (48, 70.2) CI (50, 69.7) AC (50, 70.2)	30	24	24	Multicomponent exercises 120 min, 1 d/wk for 12 weeks plus 6 booster sessions	120	EF, PS, attention, memory, language	Structured exercise program center-based:2 d/wk for 12 weeks; 12 wk. home-based sessions; number not clear.	180	Strength, balance	None	Separate	Mixed	AC: Leisure activities	**Cognitive**: global cognition, memory, language, attention, EF
Ngandu et al. (2015)	Finland	1,190	EI-CI (591, 69.5) AC (599, 69.2)	538	96	-	10 group-based sessions on memory and reasoning strategies, and 2 x 6 months 72 (10–15 min, 3 d/wk) home-based, computerized training.	37	EF, PS, memory, attention	Center-based supervised, structured, and individualized exercise program 3–5 d/wk	not clear	Fitness, strength	Not clear	Separate	Mixed	AC: Health education	**Cognitive**: global cognition, EF, PS, memory
Nilsson et al. (2020)	Sweden	73	EI-CI (25, 70.3) CI (21, 70.9) EI (27, 70.3)	30	12	-	Computerized working memory training 2.5 d/wk	75	EF	Supervised interval training on stationary bikes at 65–75% HRmax. 2.5 d/wk	90	Fitness	Moderate	Sequential	Group	-	**Cognitive**: EF, PS, memory, language
Nishiguchi et al. (2015)	Japan	48	EI-CI (24, 7) PC (24, 73.5)	12	12	-	Verbal exercises, psychomotor 1 d/wk	60	EF, language	Group classes with music soundtrack 1 d/wk	90	Fitness, strength	Not clear	Simultaneous	Group	-	**Cognitive**: global cognition, memory, EF **Physical**: fitness, balance, strength
Nocera et al. (2020)	USA	37	EI-CI (13, 72.1) EI (12, 69.5) CI-EC (12, 7)	36	12	-	Computerized cognitive training (Mindfit) 3 d/wk	60	EF and “other processes”	Supervised stationary bicycle riding at 50 to 75% HRmax. 3 d/wk	135	Fitness	Moderate	Sequential	Group	EC: Stretching	**Cognitive**: EF, memory, language, PS **Physical:** fitness, balance (gait)
Norouzi et al. (2019)	Iran	40	EI-CI (20, 68.5) AC (20, 68.1) EC not incl.	12	4	12	Verbal and visual tasks 3 d/wk	210	EF, memory	Supervised strength training using an isokinetic exercise device. 3 d/wk	210	Strength	None	Simultaneous	Group	AC: group discussions	**Cognitive**: EF, memory **Physical**: strength
Oswald et al. (2006)	Germany	196	EI-CI (24, 79.5) EI (29, 79.5) CI (46, 79.5) PC (97, 79.5)	30	48	48	Multicomponent exercises 1 d/wk	45	Memory, attention, PS	Supervised exercise program including gymnastics, dance, games, tennis skills, etc. 1 d/wk	45	Balance, fitness	Low	Sequential	Group	-	**Cognitive**: composite score from multiple test-domains **Physical**: composite score from multiple test-domains
Phirom et al. (2020)	Thailand	39	EI-CI (19, 70.2) PC (20, 69.4)	36	12	-	Exergames 3 d/wk	180	EF, memory, attention	Center-based exergames (Xbox) 3 d/wk	180	Fitness, balance	Low	Simultaneous	Group		**Cognitive**: global cognition **Physical**: balance, strength
Pieramico et al. (2012)	Italy	30	EI-CI (15, 67.5) PC (15, 67.5)	144	24	-	Home-based cognitive activities 5 d/wk, and group activities 120 min, twice a month	300	Not clear	Structured home-based walking and dancing 2 d/wk	120	Fitness	Low	Separate	Mixed	-	**Cognitive**: global cognition, EF, memory, language, PS
Rahe et al. (2015a)	Germany	45	EI-CI (25, 68.4) CI (20, 67.6)	14	7	-	Multicomponent exercises 2 d/wk	140	Memory, EF, attention	Group classes and home exercises (walking, taking stairs) 2 d/wk	40	Fitness, balance, strength	Low	Sequential	Group	-	**Cognitive**: global cognition, memory, EF, language, attention **Physical**: fitness, strength
Rahe et al. (2015b)	Germany	30	EI-CI (15, 67.1) CI (15, 66.3)	13	6.5	48	Multicomponent exercises 2 d/wk	190	Memory, EF, attention	Supervised structured exercise program 2 d/wk	40	Fitness, balance, strength	Low	Sequential	Group	-	**Cognitive**: global cognition, EF, language, attention
Raichlen et al. (2020)	USA	51	EI-CI (12, 67.7) EI (17, 68.1) CI (10, 66.4) AC (12, 69.3)	36	12	-	Computerized cognitive training 3 d/wk	90	EF, PS, memory,	Supervised stationary bicycle riding at 40–80% HRmax 3 d/wk	90	Fitness	Moderate	Simultaneous	Group	AC: watching videos	**Cognitive**: EF **Physical**: balance (gait)
Romera-Liebana et al. (2018)	Spain	352	EI-CI (176, 77.2) PC (176, 77.4)	24	12	18	Multicomponent memory and verbal training 2 d/wk	180 (6 wks)	Memory, language	Supervised structured exercise program 2 d/wk	120 (6 wks)	Fitness, balance, strength	Not clear	Separate	Group	Nutritional supplement	**Cognitive**: memory, language **Physical**: fitness, balance, strength
Salazar-González et al. (2015)	Mexico	286	EI-CI (143, 71) PC (143, 74)	36	12	-	Verbal exercises, psychomotor tasks 3 d/wk	60	EF	Supervised structured exercise program 3 d/wk	180	Fitness, balance, strength	Low	Simultaneous	Group	-	**Cognitive**: EF **Physical**: Balance (gait)
Schoene et al. (2013)	Australia	32	EI-CI (15, 77.5) PC (17, 78.4)	22	8	-	Home-based exergame (Stepmania) 1.5 d/wk	30	EF	Home-based exergames involving step exercises 1.5 d/wk	30	Fitness	Low	Simultaneous	Individual	-	**Cognitive**: PS, EF **Physical:** balance (+postural stability), strength
Schoene et al. (2015)	Australia	81	EI-CI (39, 82.7) PC (42, 81)	48	16	-	Home-based exergames (Stepmania, Trail-Stepping, Stepper, Tetris), 3 d/wk	60	EF, PS, attention	Home-based exergames involving step exercises 3 d/wk	60	Fitness	Low	Simultaneous	Individual	-	**Cognitive**: EF, PS
Shah et al. (2014)	Australia	172	EI-CI (44, 67.2) EI (42, 67.4) CI (51, 66.6) PC (35, 69.1)	160	16	-	Computerized cognitive training (Posit Science) 5 d/wk	300	Not clear	Supervised structured exercise program 5 d/wk	250	Fitness, strength	Low	Sequential	Individual	-	**Cognitive**: memory, language, PS, attention, EF **Physical:** fitness, strength
Shatil (2013)	USA	122	EI-CI (29, 79) EI (31, 79) CI (33, 80) AC (29, 81)	96	16	-	Computerized cognitive training (CogniFit) 3 d/wk	120	EF, PS, attention, language, memory	Supervised structured exercise program (FitnessForever™) 3 d/wk	135	Fitness, strength	Low	Separate	Group	AC: book reading	**Cognitive**: Memory, attention, EF, PS
Takeuchi et al. (2020)	Japan	93	EI-CI (30, 68) CI (30, 68.8) EI (33, 69.3)	36	12	-	Computerized cognitive training (Brain Age, Nintendo) 3 d/wk	180	EF	Center-based supervised stationary bike riding at 40–50% HRmax 3 d/wk	90	Fitness	Low	Simultaneous	Not clear	-	**Cognitive**: Memory, attention, EF, PS, language
Teixeira et al. (2013)	Brazil	41	EI-CI (21, 68.2) PC (20, 67.9)	48	16	-	Square stepping 3 d/wk	120	Attention, memory, EF	Supervised, structured square stepping exercises 3 d/wk	120	Strength, balance	None	Simultaneous	Group	-	**Cognitive**: global cognition, EF, memory, attention, PS
Theill et al. (2013)	Switzerland	51	EI-CI (18, 72.4) CI (12, 73.3) PC (21, 70.9)	20	10	-	Computerized working-memory training 2 d/wk	60	EF	Supervised center-based-treadmill walking at 60–80% HRmax 2 d/wk	80	Fitness	Moderate	Simultaneous	Not clear	-	**Cognitive**: attention, memory, EF, PS **Physical**: balance (gait)
van Het Reve and de Bruin (2014)	Switzerland	145	EI-CI (69, 81.1) EI (76, 81.9)	84	12	-	Computerized cognitive training (CogniPlus) 3 d/wk	30	Attention	Progressive strength training and balance training. 2 d/wk	80	Balance, strength	None	Sequential	Not clear	-	**Cognitive**: EF, attention **Physical:** balance (gait), fitness
Wollesen et al. (2017)	Germany	83	EI-CI^b^ (30, 69.8) PC^b^ (18, 72.7) EI-CI^c^ (15, 72.2) PC^c^ (20, 72)	12	12	-	Psychomotor tasks 1 d/wk	60	EF, attention	Supervised walking exercises 1 d/wk	60	Fitness	Low	Simultaneous	Group	-	**Cognitive**: EF **Physical**: balance (gait), fitness
Wongcharoen et al. (2017)	Thailand	45	EI-CI (15, 71.9) EI (15, 73.5) CI (15, 72.4) CI dual-task not incl.	12	4	-	Cognitive tasks 3 d/wk	180	Attention, memory, language	Home-based stance and gait activities 3 d/wk	180	Balance	None	Simultaneous	Mixed	-	**Cognitive**: EF, language **Physical**: balance (gait)
Yokoyama et al. (2015)	Japan	25	EI-CI (12, 74.2) EI (13, 74.2)	48	12	-	Verbal exercises, psychomotor tasks 3 d/wk	180	EF	Supervised structured exercise program 3 d/wk	180	Fitness, balance	None	Simultaneous	Group	-	**Cognitive**: global cognition, PS **Physical:** strength, fitness, balance
You et al. (2009)	South Korea	13	EI-CI (8, 68.3) EI-CC (5, 68)	18	6	-	Verbal exercises 3 d/wk	90	EF, memory	Supervised fast walking 3 d/wk	90	Fitness	Moderate	Simultaneous	Not clear	CC: Music	**Cognitive**: memory **Physical:** balance (gait)
Yu et al. (2021)	China	347	EI-CI^d^ (117, 64.7) EI-CC (114, 64) EI-CI^e^ (116, 64)	24	12	-	Computerized cognitive training (Brainastic) 2 d/wk	60	EF, memory, attention	Aerobic circuit and resistance training 2 d/wk	120	Fitness, strength	Moderate	Sequential	Group	CC: DVDs	**Cognitive**: global cognition, memory

Outcome measures varied across the studies, with most of the studies assessing several cognitive functions, such as attention, switching, executive functions, processing speed, memory, and global cognition (see Supplementary Table 5), as well as physical outcomes, such as strength, endurance, frailty, gait, balance, risk of falls, functional mobility or VO_2_max.

#### Analysis of Bias

**Figure 2:**
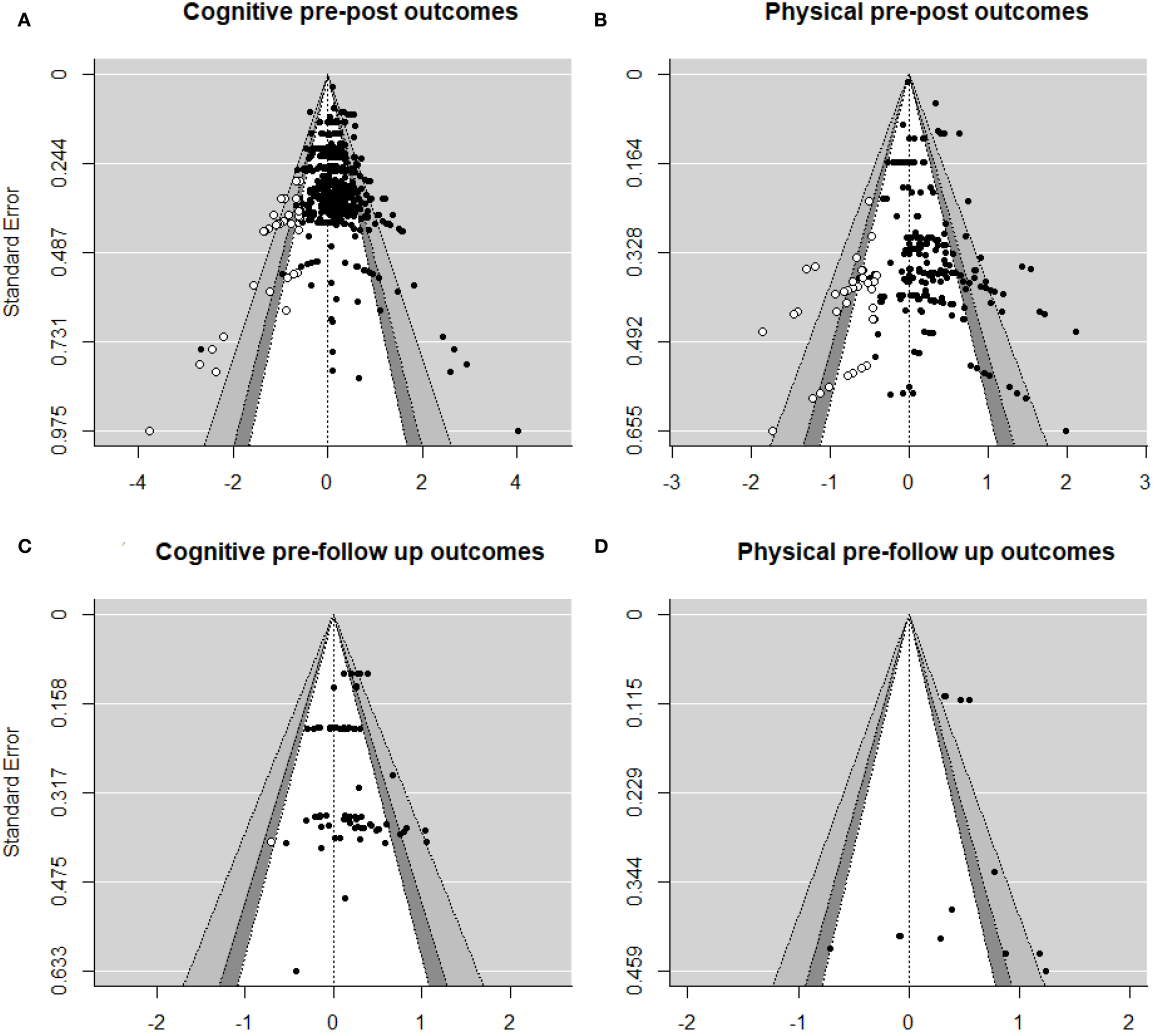
Funnel plots with ES on the X-axis and standard error of the ES on the Y-axis for the estimated summary effects of **(A)** cognitive, **(B)** physical pre-post outcomes, **(C)** cognitive, and **(D)** physical pre-follow up outcomes.

A visual inspection of the funnel plot corresponding to cognitive pre-post outcomes [number of effect sizes (*k*) = 507] revealed asymmetry with larger effect sizes on the right lower side of the plot, which was confirmed by the Egger's regression test (*z* = 4.108, *p* < 0.001, β = −0.024, 95*% CI* [−0.112, 0.064]). This test is identical to regressing effect sizes on standard errors, where weights are inversely proportional to the variance of effect sizes. In the Egger's test a significant positive intercept means that smaller studies with less precision are associated with larger effects. The trim-and-fill method estimated that to restore symmetry are necessary to add 32 ES to the left side of the plot, which would reduce the estimated summary effect to 0.114 (*p* < 0.001, 95*% CI* [0.083, 0.145]). Even though smaller studies produced the largest effect sizes, the standard errors of effect sizes were represented uniformly in a range from 0.244 to 0.975, suggesting that the underrepresentation of negative results was not only a question of small-study effects (i.e., higher standard errors) but occurred in smaller as well as in larger samples (see Figure 2A). The results of the fail-safe tests indicated that it would need 21,678 ES (based on Rosenberg's approach) or 30.933 ES (following Rosenthal's approach) to increase the *p*-value of an overall ES of 0.145 to above 0.05. According to Owen's approach, 507 ES would be necessary to reduce the average ES from 0.194 to 0.097.

Regarding physical functions (*k* = 203), the funnel plot also suggested an asymmetry skewed to the right. Again, Egger's test was significant (*z* = 4.225, *p* < 0.001, β = 0.017, 95*% CI* [−0.103, 0.136]), and the trim-and-fill method estimated that 27 (*p* < 0.001, 95*% CI* [0.113, 0.234]) ES should be added to restore the symmetry of the funnel plot, reducing the estimated summary effect to 0.174 (Figure 2B). In this case, the imputed effect sizes for the funnel plot to be symmetric were in a lower range of standard errors, indicating that especially negative results from studies with lower precision were needed to restore the symmetry. However, compared to the cognitive outcomes, the main amount of ES was in the middle of the plot, suggesting fewer studies with large samples in physical outcomes than in cognitive outcomes. To reduce the significance of an overall ES of 0.091 to a P level above 0.05, 13,326 ES would be needed taking Rosenthal's approach, or 3,540 ES taking Rosenthal's approach. According to Owen's approach, it would be necessary 203 additional ES to reduce the ES from 0.316 to 0.158.

In the case of cognitive pre/follow-up outcomes (*k* = 73) (Figure 2C), we detected no asymmetry, which was confirmed by a non-significant Egger's test (*z* = 0.176, *n*.*s*., β = 0.166, 95*% CI* [0.056, 0.277]). The trim-and-fill method estimated that only one ES (*p* < 0.001, 95*% CI* [0.12, 0.223]) would be necessary to restore the symmetry of the funnel plot. According to Rosenberg, it would need 871 ES, and according to Rosenthal, 970 ES, to increase the P level of an average ES of 0.178 to above 0.05. Orwin's approach estimated that 73 ES would be necessary to add to reduce an average ES of 0.19–0.09.

Regarding physical pre/follow-up outcomes (Figure 2D), the results of the bias analysis should be taken with caution because of the reduced dataset (*k* = 13). Egger's test did not detect any asymmetry (*z* = 0.117, *n*.*s*, β = 0.408, 95*% CI* [0.212, 0.6]), and the fail-safe calculations indicated that it would be necessary 225 (Rosenberg) or 218 (Rosenthal) ES to reduce the statistical significance of an ES of 0.416 to above 0.05. According to Owen's approach, it would require 13 ES to reduce the estimated ES of 0.427 to 0.214. The trim-and-fill method estimated that no ES had to be added to restore the symmetry (*n*.*s*., 95*% CI* [0.309, 0.525]).

### Overall Effect Size

**Figure 3:**
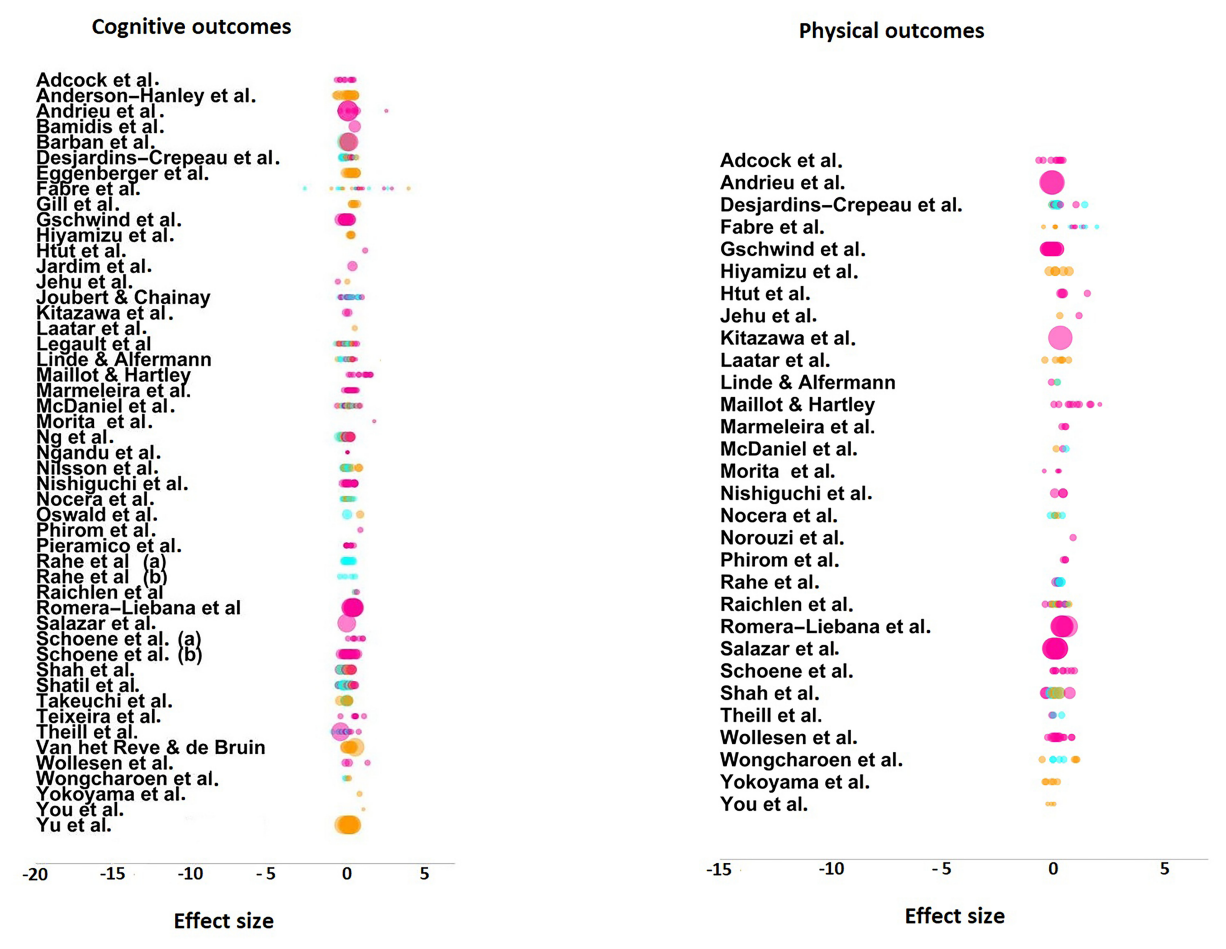
Dot-plot figures for effect sizes for cognitive outcomes and physical outcomes by primary studies. Pink dots represent combined training *vs*. control, blue dots represent combined *vs*. single cognitive training, and orange dots, combined *vs*. single physical training. The size of the dot indicates the inverse of the ES variance scaled and represents the precision of the ES.

Figure 3 displays the summary effect of pre-post cognitive and physical outcomes by study. The estimated summary effect across all studies (*n* = 50) for pre-post comparison of cognitive outcomes (*k* = 507) was *g* = 0.22 (*p* < 0.001, 95% CI [0.152, 0.289]) (see Table 2). The summary effect of standardized mean differences differed significantly across groups [*F*_(2, 504)_ = 11.588, *p* < 0.001] and was highest for combined *vs*. control comparisons (*g* = 0.275, *p* < 0.001, 95*% CI* [0.201, 0.359]), followed by combined *vs*. single physical training (*g* = 0.21, *p* < 0.001, 95*% CI* [0.128, 0.291]). On the other hand, the summary effect of cognitive outcomes for combined *vs*. single cognitive training was similar (*g* = 0.083, n.s., 95% CI [-0.001, 0.169]). The summary effect for physical outcomes (*k* = 190) was 0.285 (*p* < 0.001, 95*% CI* [0.192, 0.378]). Combined training produced a superior effect in all comparisons [*F*_(2, 187)_ = 0.886, *n*.*s*.], which was highest when compared to single cognitive training (*g* = 0.33, *p* < 0.001, 95*% CI* [0.171, 0.489]), followed by the comparison with control groups (*g* = 0.30, *p* < 0.001, 95*% CI* [0.198, 0.412]), and single physical training (*g* = 0.218, *p* < 0.01, 95*% CI* [0.073, 0.363]).

**Table 2:** Summary effect of pre-post and pre-follow up comparisons of pooled cognitive and physical differences of effect sizes, respectively.

	**Comparison**	**Level 2 variance (%)**	**Level 3 variance (%)**	**QE**	**# Studies**	**# ES**	**Mean difference in ES [95% CI]**
Cognitive functions	Pre-post	0.005 (4.9)	0.041 (36.991)^***^	791.173^***^	49	507	0.22 [0.152, 0.289]^***^
	Pre-follow up	0.000 (5.326e-09)	0.026 (35.619)^**^	71.335	10	73	0.205 [0.073, 0.338]^**^
Physical functions	Pre-post	0.000 (7.649e-08)	0.045 (54.278)^***^	424.825^***^	30	190	0.285 [0.192, 0.378]^***^
	Pre-follow up	0.003 (7.842)	0.000 (1.021e-07)	21.622^*^	4	13	0.417 [0.297, 0.538]^***^

# *Studies, Number of studies; #ES, Number of effect sizes; ES, Hedges' g; CI, Confidence interval; Level 2 variance, Variance in effect sizes within studies; Level 3 variance, Variance in effect sizes between studies; %, Proportion of the total variance of effect sizes attributed to this level; QE, test for heterogeneity in all effect sizes in the data set*.

* *p < 0.05*;

** *p < 0.01*;

*** *p < 0.001*.

Regarding cognitive pre-follow-up outcomes (*k* = 73), we found a summary effect of 0.205 (*p* < 0.01, 95*% CI* [0.073, 0.338]). The differential effect of combined training differed across group comparison (*F*_(2, 70)_ = 4.093, *p* < 0.05), and was highest when compared to control groups (*g* = 0.31, *p* < 0.01, 95*% CI* [0.107, 0.513]), followed by single physical training (*g* = 0.239, *p* < 0.05, 95*% CI* [0.037, 0.442]). Combined training did not show superior effects at follow-up when compared to single cognitive training (*g* = 0.073, *n*.*s*., 95*% CI* [−0.128, 0.275]). Only 4 studies reported results of physical pre-follow-up assessments. Also, no ES was reported for a combined *vs*. single cognitive comparison. For combined *vs*. single physical training and control group comparisons, the summary effect was 0.417, with no significant group differences (*F*_(2, 11)_ = 1.462, *n*.*s*.). Nonetheless, due to the low number of effect sizes (*k* = 13), this result should be interpreted with caution. Combined training produced a significant superior effect when compared to control groups (*g* = 0.584, *p* < 0.01, 95*% CI* [0.199, 0.968]), however, the comparison with single physical training did not reach statistical significance (*g* = 0.243, *p* = *n*.*s*., 95*% CI* [−0.259, 0.745]). Given the low number of ES, we did not analyze the follow-up results by functions, as most categories x group combination contained less than three ES.

According to Hunter and Schmidt (1990), heterogeneity can be regarded as substantial if sampling variance (variance explained by the specific participants sampled in the experiment) is below 75%. This criterion was achieved for both of our main conditions (cognitive and physical pre-post ES), justifying our three-level meta-analytic approach. In both cases, the three-level model provided a significantly better fit compared to a two-level model with level 3 heterogeneity constrained to zero, as indicated by the likelihood ratio test (LRT) $\left(cognitive:\;\chi_{1}^{2}=7.554,\;p<0.001,\;physical:\;\chi_{1}^{2}=47.909,\;p<0.001\right)$. Also, the Akaike (AIC) and Bayesian Information Criterion (BIC) were lower for the three-level models, indicating improved model fits. On the other hand, we found in both conditions (cognitive and physical pre-post ES) a relatively high variance attributable to the estimated sampling variance and the between-study variability, but very little (4.9% for cognitive pre-post outcomes), or none of the proportion (for physical pre-post outcomes) explained by the within-study level. The low level 2 variance suggests that the differences in effect sizes within each study were consistent across the comparison groups. On the other hand, approximately half of the studies included only one type of comparison and, for the other half, two or more types of comparisons (see Table 1 with the descriptive data). Thus, the source of the level 3 variance could be attributable to a combination of the differential treatment effects (e.g., combined *vs*. control from one study, combined *vs*. single cognitive from another study, etc.), and different effect size magnitudes across studies (e.g., combined *vs*. control from several studies).

### Moderator Analyses

#### Pre-post Training Effects by Cognitive Function

We analyzed the training effects on seven categories of cognitive functions (executive functions, attention, memory, language, processing speed, global functioning, and composite scores) using REML as the estimation method. These seven categories were crossed with the standardized mean difference of effect sizes of group comparisons (combined *vs*. single cognitive, combined *vs*. single physical, and combined *vs*. control). Their means, confidence intervals, statistical significance, as well as QE-values as a test of heterogeneity for all effect sizes, and the level 2 and level 3 variances are displayed in Table 3.

**Table 3:** Results of moderator analyses for pre-post comparisons between combined training vs. control, cognitive or physical single for cognitive and physical outcomes.

**Outcomes**	**Level 2 variance (%)**	**Level 3 variance (%)**	**Omnibus test^**a**^**	**QE**	**Comparison groups**	**# Studies**	**# ES**	**Mean difference in ES [95% CI]**
**Cognitive functions**								
Executive functions	0.00 (7.811e-08)	0.024 (22.971)^***^	*F*_(2, 161)_ = 0.42, *p* = 0.657	189.618	Combined vs. control	21	80	0.2 [0.103, 0.297]^***^
					Combined vs. cognitive	13	44	0.144 [0.021, 0.267]^*^
					Combined vs. physical	14	40	0.199 [0.081, 0.316]^***^
Memory	0.000 (4.286e-08)	0.039 (36.098)^***^	*F*_(2, 138)_ = 5.051, *p* = 0.008	251.221^***^	Combined vs. control	19	50	0.204 [0.088, 0.321]^**^
					Combined vs. cognitive	15	43	0.007 [-0.119, 0.134]
					Combined vs. physical	17	48	0.117 [-0.017, 0.256]^*^
Attention	0.019 (17.262)	0.02 (18.141)	*F*_(2, 47)_ = 5.176, *p* = 0.009	71.632^*^	Combined vs. control	10	28	0.197 [0.038, 0.358]^*^
					Combined vs. cognitive	8	11	−0.166 [-0.383, 0.051]
					Combined vs. physical	7	11	0.19 [-0.015, 0.396]
*Language*	0.00 (7.64e-09)	0.036 (45.287)^***^	*F*_(2, 31)_ = 3.387, *p* = 0.047	30.875^*^	Combined vs. control	6	11	0.305 [0.123, 0.487]^**^
					Combined vs. cognitive	9	11	−0.008 [-0.201, 0.186]
					Combined vs. physical	9	12	0.08 [-0.102, 0.264]
Speed	0.00 (1.312e-08)	0.104 (54.037)^***^	*F*_(2, 88)_ = 3.481, *p* =0.035	148.492^**^	Combined vs. control	15	47	0.308 [0.129, 0.486]^***^
					Combined vs. cognitive	9	19	0.046 [-0.163, 0.256]
					Combined vs. physical	14	25	0.258 [0.069, 0.447]^**^
Global cognition	0.000 (1.211e-08)	0.153 (86.725)^*^	*F*_(2, 15)_ = 1.655, *p* = 0.224	44.504^***^	Combined vs. control	8	10	0.525 [0.172, 0.877]^**^
					Combined vs. cognitive^a^	1	1	NA
					Combined vs. physical	2	7	−0.048 [-0.621, 0.524]
Composite scores	0.052 (39.027)	0.019 (14.62)	*F*_(2, 6)_ = 2.884, *p* = 0.133	16.743^*^	Combined vs. control	5	4	0.392 [-0.017, 0.8]
					Combined vs. cognitive^a^	3	3	NA
					Combined vs. physical^a^	2	2	NA
**Physical functions**								
Fitness	0.00 (2.848e-08)	0.059 (61.28)^***^	*F*_(2, 62)_ = 1.917, *p* = 0.156	176.29^***^	Combined vs. control	16	33	0.242 [0.075, 0.409]^**^
					Combined vs. cognitive	8	18	0.338 [0.105, 0.571]^**^
					Combined vs. physical	9	15	0.064 [-0.185, 0.313]
*Balance*	0.00 (2.757e-08)	0.026 (30.205)^***^	*F*_(2, 92)_ = 0.192, *p* = 0.826	130.952^**^	Combined vs. control	17	58	0.273 [0.149, 0.396]^***^
					Combined vs. cognitive	4	12	0.196 [-0.052, 0.444]
					Combined vs. physical	9	25	0.229 [0.045, 0.413]^*^
*Strength*	0.037 (18.584)	0.092 (46.711)	*F*_(1, 27)_ = 0.266, *p* < 0.768	71.739^***^	Combined vs. control	12	20	0.372 [0.103, 0.642]^**^
					Combined vs. cognitive	3	5	0.463 [-0.081, 1.007]
					Combined vs. physical	5	7	0.227 [-0.177, 0.632]

a *ES differences were only calculated for analyses with more than 3 ES*.

# *Studies, Number of studies; #ES, Number of effect sizes; mean ES, mean Hedges' g; CI, Confidence interval; Level 2 variance, Variance in effect sizes within studies; Level 3 variance, Variance in effect sizes between studies; %, Proportion of the total variance of effect sizes attributed to this level; QE, test for heterogeneity in all effect sizes in the data set. Omnibus-test of all coefficients in the model (excluding the intercept)*.

* *p < 0.05*;

** *p < 0.01*;

*** *p < 0.001*.

In executive functions, combined training achieved superior effects in comparison to control groups (*g* = 0.201, *p* < 0.001), single physical (*g* = 0.199, *p* < 0.01), and single cognitive training (*g* = 0.144, *p* < 0.05). In memory and speed, combined training produced superior training effects compared to control groups (*g* = 0.204, *p* < 0.001 and *g* = 0.308, *p* < 0.001, for memory and speed, respectively), and to single physical training (*g* = 0.137, *p* < 0.05 and *g* = 0.258, *p* < 001, for memory and speed, respectively), whereas no significant differences were found in these categories when compared to single cognitive training (*g* = 0.007, n.s., and *g* = 0.046, n.s., for memory and speed, respectively). In attention, language, and global cognition, combined training only produced superior effects when compared with control groups (*g* = 0.197, *p* < 0.05, *g* = 0.305, *p* < 0.01 and *g* = 0.525, *p* < 0.01, for attention, language, and global cognition, respectively). No other statistically significant differences were found.

#### Pre-post Training Effects by Physical Function

We analyzed the effect of the three training categories on the physical functions assessed in the original studies (balance, fitness, and strength), crossed with the type of training (combined, cognitive, and physical). Combined training showed significantly superior effects in comparison to control groups in fitness (*g* = 0.242, *p* < 0.01), balance (*g* = 0.273, *p* < 0.001), as well as in strength (*g* = 0.372, *p* < 0.01). Furthermore, combined training showed an advantage over single physical training in balance (*g* = 0.229, *p* < 0.05), and over single cognitive training in fitness (*g* = 0.338, *p* < 0.01). No other group comparisons resulted statistically significant.

#### Design, Study Quality, and Sample Characteristics

We identified several study characteristics that could potentially modify the training outcomes (see Supplementary Tables S5, S6 for detailed information).

*Combinatory mode*. Combined physical and cognitive training could be performed simultaneously (cognitive and physical training was performed at the same time), sequential (one after another) or separate (on different days). Our results indicated that the largest training effects in executive functions were produced by simultaneous training (*g* = 0.208, *p* < 0.001), followed by training on separate days (*g* = 0.175, *p* < 0.05). Sequential training did not produce a significant effect size in this case *g* = 0.157, *p* > 0.05). In attention, simultaneous (*g* = 0.144, *p* < 0.05), as well as sequential training (*g* = 0.286, *p* < 0.05), had an advantage over training on separate days (*g* = −0.139, *n*.*s*.) (*F*_(2, 47)_ = 4.483, *p* < 0.05). In speed, simultaneous training was related with an effect of 0.293 (*p* < 0.01). Neither sequential training (*g* = −0.007, *n*.*s*.), nor training on separate days (*g* = 0.138, *n*.*s*.) were associated with significant training gains. In global cognition, simultaneous training resulted significantly superior (*g* = 0.56, *p* < 0.05) to sequential (*g* = 0.156, *n*.*s*.) and separate training (*g* = 0.161, *n*.*s*.) (*F*_(2, 15)_ = 41.064, *p* < 0.001.). As for the physical outcomes, only simultaneous training produced a significant effect size in outcomes that measured balance (*g* = 0.259, *p* < 0.001) and strength (*g* = 0.223, *p* < 0.05). No other significant differences were found.

##### Aerobic vs. Non-aerobic Training

Aerobic intensity was classified either based on objective measures provided by the authors (HRmax, velocity, etc.), or based on the description of the physical activities. Low to non-aerobic exercise, such as slow walking, strength, or balance training were classified as non-aerobic. Moderate to high aerobic intensity, such as walking at a fast pace or running were classified as aerobic. Gains in executive functions were larger for aerobic (*g* = 0.20, *p* < 0.001) than for non-aerobic exercise (*g* = 0.138, *p* < 0.01), even though the difference did not reach statistical significance (*F*_(2, 147)_ = 0.732, *n*.*s*.). Aerobic exercise (*g* = 0.279, *p* < 0.01) was related to more improvement in attention than non-aerobic exercise (*g* = 0.032, *n*.*s*.) (*F*_(1, 48)_ = 5.084, *p* < 0.05), whereas non-aerobic exercise produced larger effects in speed (*g* = 0.202, *p* < 0.05), and global cognition (*g* = 0.508, *p* < 0.01). In physical categories, as could be expected, aerobic training was related to higher gains in fitness (*g* = 0.257, *p* < 0.01) than non-aerobic training (*g* = 0.059, *n*.*s*.), and non-aerobic exercise produced larger gains in balance (*g* = 0.272, *p* < 0.001 and *g* = 0.182, n.s., for non-aerobic and aerobic, respectively). No other significant results were found in this category.

##### Type of Cognitive Training

Cognitive training was categorized as computer training (commercial videogames or tailor-made computer tasks), interactive training (dual-task paradigms in which the cognitive training part is intrinsically associated with a motor response, as in exergames, square stepping, etc.), and multicomponent training (which could be either a mixture of different training modalities, such as paper-pencil tasks, computer games, verbal exercises, etc., or only verbal exercises, such as counting backward, naming words according to a given classification, etc.). Interactive training produced a significantly higher effect on speed (*g* = 0.494, *p* < 0.001) than multicomponent (*g* = 0.312, *p* < 0.05) and computer training (*g* = 0.042, n.s.) (*F*_(2, 88)_ = 4.463, *p* < 0.05). Regarding executive functions, interactive training produced an effect of *g* = 0.322 (*p* < 0.001), followed by computer training (*g* = 0.131, *p* < 0.05), and multicomponent training (*g* = 0.137, *n*.*s*.). Also, in global cognition, interactive training showed the highest effect (*g* = 0.573, *p* < 0.001). The ES from the interactive training type stemmed in 90% of the cases from combined *vs*. control comparisons, because the cognitive activity is intrinsically associated with a motor response, so that it is impossible to perform the cognitive part separately. To confirm that the differences in training gains as a function of cognitive training type were not influenced by the underlying group comparisons, we repeated the analysis in executive functions and speed only for those cases that had been computed from combined *vs*. control comparisons. In executive functions, only interactive training achieved a significant ES (*g* = 0.318, *p* < 0.001), whereas the training gains associated with computer training (*g* = 0.114, *n*.*s*.), and multicomponent training (*g* = 0.136, *n*.*s*.) were not significant. The same occurred with speed, with interactive training achieving a medium ES (*g* = 0.475, *p* < 0.001), in contrast with non-significant gains in the case of computer (*g* = 0.055, *n*.*s*.), and multicomponent training (*g* = 0.34, *n*.*s*.). On the other hand, multicomponent training was related with the highest effects in memory (*g* = 0.196, *p* < 0.05) and language (*g* = 0.228, *p* < 0.05), without reaching the other modalities statistical significance. In physical outcomes, interactive and multicomponent training were related with significant effects on balance (*g* = 0.301, *p* < 0.001 and *g* = 0.269, *p* < 0.01, for interactive and multicomponent training, respectively). Interactive and multicomponent training were also related with significant improvements in fitness (*g* = 0.385, *p* < 0.01 *and g* = 0.288, *p* < 0.01, *for interactive and multicomponent, respectively*). Furthermore, interactive training was related with a significant effect in strength (*g* = 0.411, *p* < 0.05).

##### Setting

The training could either be performed in groups, individually, or in a mixed setting (some sessions group based, and others conducted individually). Group setting produced significant effects in all cognitive categories as opposed to individual or mixed setting. In executive functions, only the ES of group setting (*g* = 0.162, *p* < 0.001) and individual training (*g* = 0.151, *p* < 0.05) resulted significant. Group training was related with an effect of *g* = 0.182 (*p* < 0.01) for memory, *g* = 0.189 (*p* < 0.05) for attention, and *g* = 0.482 (*p* < 0.05) for global cognition. In language and speed, mixed training produced superior effects (*g* = 0.333, *p* < 0.05, *and* *g* = 0.348, *p* < 0.05, for language and speed, respectively), than group training (*g* = 0.207, *p* < 0.05 *and g* = 0.2411, *p* < 0.05, for language and speed, respectively), and in both cases significantly superior to individual training (*g* = 0.086 *and* *g* = 0.08, *n*.*s*.). Group training could not be compared to the other settings in composite scores due to insufficient ES in these categories. Regarding the physical outcomes, group setting was consistently related with significant effect sizes in all physical categories (*g* = 0.328, *p* < 0.001; *g* = 0.255, *p* < 001; *g* = 0.291, *p* < 0.05, for fitness, balance, and strength, respectively), even though individual training also showed a significant effect on balance outcomes (*g* = 0.242, *p* < 0.05).

##### Continuous Moderators

We analyzed the influence of several continuous moderators crossed with the different cognitive and physical outcome measures. We found a significant negative relationship between the number of participants and attention, suggesting that studies with smaller samples produced larger ES (β = −0.003, *p* < 0.001, CI 95% [-0.004,−0,001]). Also, studies conducted earlier achieved higher ES in fitness(β = −0.035, *p* < 0.05, *CI* 95% [−0.068, −0.002]), and studies with lower quality (β = −0.039, *p* < 0.05, *CI* 95% [−0.07, −0.008]), and higher variability in the age of participants (β = −0.11, *p* < 0.05, *CI* 95% [−0.218, −0.002]) were related to higher gains in balance. Other moderators (year of publication, quality, mean age, number and minutes of sessions, number of weeks) were not significant.

## Discussion

This systematic review and three-level meta-analysis investigated the effectiveness of combined physical and cognitive training on the cognitive and physical functions of healthy older adults. It included a total of 783 effect sizes from 50 intervention studies that investigated the differential effect of combining physical and cognitive training vs. its components alone or control groups. The included studies varied in their experimental design, and cognitive and physical activities were performed simultaneously, sequentially, or on different days, in groups or individually. Also, the cognitive training was delivered in different ways, such as via computer games, multicomponent activities, or interactively such as in exergames.

### Overall Effect Sizes

In line with previous meta-analyses (Zhu et al., 2016; Gheysen et al., 2018; Guo et al., 2020), our results revealed a small advantage of combined training on cognitive outcomes, which was maintained over time as shown by the follow-up effect. When analyzing the differential training effect by subcategories (executive functions, memory, attention, speed, language, and global cognition), combined training produced overall larger effects than control groups. In memory and processing speed, combined training also showed an advantage over single physical training. Combined training also had a small but significant advantage over single cognitive training in executive functions, whereas in the remaining cognitive functions, the effect of single cognitive training was not enlarged by the addition of physical exercise. This suggests that physical activation might act as an aggregate for the improvement of executive functions, independently of other cognitive processes. Executive functions, and their measurement, are closely related to certain aspects of attention, such as selective and divided attention. Nonetheless, we found no significant difference between combined and single cognitive training in attention, which might be related to a minor number of cases in this category.

### Training Transfer Between Cognitive and Physical Domains

In physical outcomes, combined training showed in all categories (fitness, balance, strength) an advantage over control groups. Furthermore, fitness was the only physical outcome category, in which combined training had a significant advantage over single cognitive training, indicating that combined groups, indeed, had improved their cardiovascular fitness more than single cognitive training groups. Combined training was also related to greater training gains in balance than single physical training. Given that both, combined and single physical training, performed the same type and dosage of physical exercise, and only differed in that one group additionally received cognitive training, we can speak of a transfer of cognitive training to physical balance outcomes. The transfer distance (considering near and far transfer as a continuum), depends on the degree of the interrelation of both domains. A growing body of research provides evidence of an interrelationship between cognitive processing and balance and gait in older adults (Hausdorff et al., 2005; Montero-Odasso et al., 2012; for a review, see Li et al., 2018). Especially higher cognitive functions, such as executive functions and attentional control, have been investigated in relation to postural instability, showing that, as executive functions decline with age, walking and balance become less automated and more cognitively taxing (Woollacott and Shumway-Cook, 2002). This relationship becomes especially visible in dual-task paradigms (i.e., the simultaneous performance of a cognitive task and a motor task) when older adults often tend to protect their motor functioning at the expense of the cognitive task when the situation involves a threat to balance (Schaefer and Schumacher, 2011). Consistent with the existing literature, our results confirmed that the largest training gains in executive functions were obtained when the cognitive training was delivered interactively.

### Cognitive Training Type, Combinatory Mode, and Aerobic Intensity

We considered as interactive training, dual-task paradigms in which the cognitive training part is intrinsically associated with a motor response, as in exergames or square stepping. In executive functions, interactive training more than doubled the effect achieved by computerized cognitive or multicomponent/verbal training (cognitive interventions that included verbal exercises or a mixture of different cognitive training modalities). Also, in speed measures, interactive training achieved the highest ES, which was only comparable to that obtained by multicomponent training, whereas computer training did not produce any effect on speed. In some studies, the multicomponent/verbal training was very close to interactive training (e.g., You et al., 2009; Hiyamizu et al., 2012; Jehu et al., 2017) when cognitive tasks were performed jointly with motor tasks. This suggests that the positive effect on processing speed by cognitive-physical dual tasks is boosted by situations in which cognitive challenges are intrinsically associated with functional motor responses, as it occurs in interactive training. This interpretation is also supported by our findings that simultaneous training was the only combinatory mode that was significantly related to higher gains in processing speed. Intuitively, one could postulate that processing speed would be related to cardiorespiratory fitness, in terms of more sufficient energy delivery to cerebral substrates that sustain fluid information processing. However, aerobic, and non-aerobic exercise were associated with similar training gains in processing speed. Also, in executive functions, the difference of training gains as a function of aerobic intensity was not remarkable, even though aerobic exercise was associated with slightly higher ES. Paradoxically, given the close relationship between these functions, in attention, aerobic exercise was associated with significantly higher training gains than non-aerobic exercise. Only a few studies reported and controlled the aerobic intensity with objective methods and in most cases, it was subjectively estimated. Thus, our results on the influence of the aerobic exercise intensity should be interpreted bearing in mind these limitations.

On the other hand, the mode of combining cognitive and physical activities had no significant influence on executive functions. This is an intriguing finding, as interactive training is always performed simultaneously, which, as mentioned earlier, achieved a significantly higher ES in executive functions than computer and multicomponent/verbal training. In the case of interactive training, almost 90% of the computed ES stemmed from combined *vs*. control comparisons, which produced the largest between-group differences. This could undermine to a certain degree the differences found regarding the other cognitive training types, which in many cases stemmed from combined *vs*. single cognitive comparisons. It is not possible to equate interactive cognitive interventions with single cognitive interventions as the first ones are intrinsically associated with motor responses. However, an additional analysis with only combined *vs*. control comparisons for all three cognitive training types (interactive, computer, and multicomponent) corroborated the result that interactive training was related to significantly higher effect sizes in executive functions and speed than the other two cognitive training types.

Multicomponent/verbal training produced the highest ES in language, which might be explained by the fact that in several studies in this category, the cognitive training included verbal fluency tasks (e.g., Gill et al., 2016; Wongcharoen et al., 2017; Ng et al., 2018; Romera-Liebana et al., 2018). In memory, even though interactive and multicomponent training produced similar ES, only the latter resulted statistically significant, possibly due to a higher heterogeneity in ES in the interactive training groups. Furthermore, advantageous training gains in attention were related to aerobic exercise, as well as to sequential and simultaneous training. Within the four studies with a sequential approach, 9 out of the 14 ES stemmed from one study (McDaniel et al., 2014) and originated from a tailor-made task. Thus, this finding would require replication with standardized or more common tasks. Likewise, the results in global cognition and composite scores should be interpreted with caution due to a low number of ES. In global cognition, interactive training resulted most beneficial. However, computer and multicomponent/verbal training only reported 4 and 5 ES, respectively, leading to an extremely high between-study variance (87%). On the other hand, in composite scores, multicomponent training could not be compared to the other training types, as computer training only reported two and interactive training no ES.

Regarding the physical outcomes, simultaneous training was associated with higher gains in balance and strength, reflecting the number of studies in this category that were originally designed to investigate the influence of dual-tasking on gait and balance. In line with this finding, higher gains in balance were also related to non-aerobic exercise, whereas aerobic exercise was related to gains in fitness. Interactive and multicomponent/verbal training was associated with higher effect sizes in fitness and balance, and interactive training also with higher gains in strength, whereas there was no differential effect found in computer training. This is surprising, as in more than 75% of the physical ES from the studies with computerized training, the comparison group (control and single cognitive training) had not received any physical training, as opposed to the combined training group. A tentative interpretation for this result would be that those studies that included computer training, imposed an overall lower level of physical demands on their participants so that between-group differences diminished.

### The Benefits of Group Setting

Finally, in all cognitive outcome categories, group setting, and in some categories also mixed setting, was associated with more training gains than when performing the training individually. This finding underscores the importance of social interaction in interventions with older adults. Physical improvements were also larger when participants trained in groups, indicating that social interaction contributes as a significant motivational factor for optimum attainment.

### Continuous Moderators

The analysis of continuous moderators revealed a significant negative relationship between the number of participants and ES achieved in outcomes that measured attention, with studies with lower sample sizes reporting higher ES.

None of the other moderators (quality, year of publication, mean age, number of sessions, session duration, intervention length) showed a significant influence on the cognitive results, indicating that study design and sample characteristics were overall homogenous across studies. With regards to physical outcomes, our results indicated that older studies reported higher ES in fitness and that higher variability in the mean age and lower study quality were associated with higher ES in balance outcomes.

### Publication Bias

As mentioned above, the training effects were not influenced by study quality. However, this finding needs to be interpreted with caution, as it could be influenced by publication bias (only studies with a robust study design were accepted for publication). Our results revealed that there was a risk of publication bias for training effects on cognitive, as well as on physical functions, and our estimated effect for these groups may differ from the true training effect. In particular, the large number of small-sample studies included in our analysis may have produced an overestimation of the summary effect. Nonetheless, it has been suggested that large estimates of between-study heterogeneity can cause regression asymmetry (Ioannidis and Trikalinos, 2007; Ioannidis, 2008). Indeed, our results indicated moderate to high between-study variability for cognitive and physical functions, which was larger for the latter one. The between-study heterogeneity in our analysis included on the one hand the differences in sample sizes, and on the other hand the variability between the types of comparison groups across studies. Therefore, the symmetry of the funnel plot might not constitute the most idoneous method to analyze the risk of bias. However, there is no current consensus on techniques to assess biases in three-level meta-analyses, and these results must therefore be interpreted with caution.

As far as we know, this is the first meta-analysis that controlled for equivalence of the training components in the different comparison groups. Thus, only those studies were considered for analysis, in which the physical training part of the combined group was identical to the physical exercise performed by the comparison group. Furthermore, this is the first time, that exercise intensity, as well as the type of cognitive training, are included as moderators, leading to more specific knowledge on the effects of combining both activities. Another strength of the present study is the use of a three-level meta-analytic approach to investigate the effectiveness of training in several cognitive functions and physical variables. This approach seems an effective alternative to classic meta-analysis when there is interdependence between effect sizes. Traditional univariate meta-analytic approaches assume that there is no dependence between effect sizes, and one common solution is to average the dependent effect sizes within studies into a single effect size by calculating an unweighted or—-less biased—-weighted average. When averaging or eliminating effect sizes in primary studies, there may not only be the problem of a lower statistical power due to information loss but informative differences between effect sizes are also lost and can no longer be identified in the analyses.

In sum, the results of this three-level meta-analysis indicate that even in advanced age, cognitive functioning can be improved by training, and that combined training produces a small advantage over single cognitive training on executive functions. Overall, we found evidence that a simultaneous combination of cognitive and physical activity is more effective in improving executive functions, attention, and processing speed, and that the achievement is highest when the training is performed in a social context.

### Recommendations for Future Research

Even though the present study may have contributed with more precise information on the combinatory effect of physical exercise and cognitive training on cognitive functions in healthy older adults, several issues remain unexplained and should be addressed in future research. Most importantly, to truly differentiate between mere learning effects and synergistic training benefits, it is necessary to disentangle the transfer effects and separate between near and fare transfer. Furthermore, dual-task investigations have shown that concurrent physical and cognitive activity might produce conflicts in attentional resource allocation. Therefore, future studies should control for this potential influence in their research designs, because depending on the complexity of the physical exercise, the exercise could either boost or weaken the effect of cognitive training. Lastly, an emerging field investigates the effects of immersive virtual reality (IVR) on cognition (Burin and Kawashima, 2021), where physical activity is experienced by virtual simulation. The inclusion of this type of intervention could provide interesting information in future meta-analytic research.

## Data Availability Statement

Publicly available datasets were analyzed in this study. This data can be found at: https://osf.io/582ur/?view_only=9e6a7dca659d48318faf1544cc7966e4.

## Author Contributions

JMR, SB, and JAR conceptualized the design. JAR and MM conducted the searches with the approval of the other two authors. JMR and JAR conducted the statistical analyses. SB and MM conducted the quality assessment of the reviewed articles. SB and JAR wrote the article. All authors read and approved the final version of the manuscript.

## Funding

This study was supported by grants from the Spanish Ministry of Economy and Competitiveness (grant # PSI2016-80377-R) to SB and JMR, from the Council of Madrid (S2017/BMD-3688) to JMR, and by a grant of the European Community (H2020-SC1-DTH-03-2018, grant agreement N° 826506, sustAGE) to SB. JAR was supported by a Doctoral Fellowship from the Spanish Ministry of Economy and Competitiveness (grant # BES-2017-079760).

## Conflict of Interest

The authors declare that the research was conducted in the absence of any commercial or financial relationships that could be construed as a potential conflict of interest.

## Publisher's Note

All claims expressed in this article are solely those of the authors and do not necessarily represent those of their affiliated organizations, or those of the publisher, the editors and the reviewers. Any product that may be evaluated in this article, or claim that may be made by its manufacturer, is not guaranteed or endorsed by the publisher.
