# Supplementary material for: The Effects of Combined Cognitive-Physical Interventions on Cognitive Functioning in Healthy Older Adults: A Systematic Review and Multilevel Meta-Analysis
Source: Front Hum Neurosci. 2022 Mar 24;16:838968. doi: 10.3389/fnhum.2022.838968 (PMC8987130; doi:10.3389/fnhum.2022.838968)
Supplement: Supplementary file 1 [file Data_Sheet_1.pdf]

Supplementary material

S1: Search strategy

((((“cognitive training” or “brain training” or “attention training” or “reasoning training” or “memory training” or “mental training” or “mental skills training” or “neurocognitive training” OR “executive function training” OR “attentional control training”) OR (“cognitive exercise” or “brain exercise” or “memory exercise” or “attention exercise” or “reasoning exercise”) OR (“cognitive stimulation” or “memory stimulation” or “memory enhance$” or “cognitive enhanc$” OR “executive function enhancement”) OR (“cognitive activit$” or “mental activit$”)
OR (speed and processing and training) OR mnemonic$ OR (“video game$” or videogame$ or wii or “computer game$” or “virtual reality”) OR (“cognitive intervention$” or “neurocognitive intervention$”)) AND ((exercis$ OR sport$ OR “physical fitness”) OR (“aerobic exercis$” or “aerobic train$” or “aerobic fitness” or “aerobic program$”) OR (“resistance exercis$” or “resistance train$” or “anaerobic exercis$” or “anaerobic train$” or “resistance program$”) OR (physical or aerobic or endurance or cardiorespiratory or cardiovascular or resistance or strength) OR (bicycl$ or “bike rid$” or “bicycle rid$”)))

OR

(((multimodal or multidomain or multicomponent or “multi-modal” or “multi-domain” or “multi-component” or “dual task” or “dual-task” or “tai chi” or danc$) OR (exergame$ or “active video game$” or “active videogame$” or kinect or “active play” or “interactive video”)) AND (cognitive adj2 physical) AND (cognition or cognitive or memory or executive function$ or “executive control” or attention or visuospatial or “processing speed” or language)))

AND (“older adults” or elder$ or senior$ or adult$ or older or ag?ing)

Table S2. Assessment tools used in the included studies and their classification into cognitive (executive functions, memory, speed, attention, global cognition, and composite scores), and physical functions (fitness, balance, and strength).			
**Authors**	**Year**	**Test**	**Function**
Adcock et al	2020	2min stepping test	fitness
Adcock et al	2020	30 s chair rises test	strength
Adcock et al	2020	Cycle duration CV [%]	balance
Adcock et al	2020	Digit span backward score	executive
Adcock et al	2020	Digit span forward	memory
Adcock et al	2020	Digit span forward score	memory
Adcock et al	2020	Extended balance test of SPPB	balance
Adcock et al	2020	Gait Speed mean [m/s]	fitness
Adcock et al	2020	Stride length CV [%]	balance
Adcock et al	2020	Stride length mean [m]	balance
Adcock et al	2020	TMT A -s	speed
Adcock et al	2020	TMT B-s	executive
Adcock et al	2020	Toe clearance CV [%]	balance
Adcock et al	2020	Toe clearance mean [cm]	balance
Adcock et al	2020	Victoria 3-2	executive
Adcock et al	2020	Victoria Stroop 1 - time	speed
Adcock et al	2020	Victoria Stroop 2 - time	speed
Adcock et al	2020	Victoria Stroop 3 - time	executive
Anderson-Hanley et al	2012	Clock drawing	global
Anderson-Hanley et al	2012	Color trail test	executive
Anderson-Hanley et al	2012	COWAT-categories	language
Anderson-Hanley et al	2012	COWAT-total	language
Anderson-Hanley et al	2012	Digit Span backward	executive
Anderson-Hanley et al	2012	Figure copy	global
Anderson-Hanley et al	2012	Figure copy delayed	global
Anderson-Hanley et al	2012	Letter Digit Symbol Test	attention
Anderson-Hanley et al	2012	RAVLT delayed	memory
Anderson-Hanley et al	2012	RAVLT immediate	memory
Anderson-Hanley et al	2012	RAVLT_5trials	memory
Anderson-Hanley et al	2012	Stroop	executive
Andrieu et al	2017	Category Naming Test	language
Andrieu et al	2017	Composite z score	composite
Andrieu et al	2017	COWAT	language
Andrieu et al	2017	DSST	speed
Andrieu et al	2017	Free and Cued Selective Reminding	memory
Andrieu et al	2017	Gait speed	fitness
Andrieu et al	2017	MMSE	global
Andrieu et al	2017	MMSE orientation	global
Andrieu et al	2017	SPPB	fitness
Andrieu et al	2017	TMT-A	speed
Andrieu et al	2017	TMT-B	executive
Andrieu et al	2017	visual analogue scale	memory
Bamidis et al	2015	Composite z score	composite
Barban et al	2017	RAVLT delayed	memory

Desjardins-Crepeau et al	2016	6-min walk	fitness
Desjardins-Crepeau et al	2016	Baddeley dual task - single	speed
Desjardins-Crepeau et al	2016	Baddeley dual-task interfern index	executive
Desjardins-Crepeau et al	2016	Chair stand test	strength
Desjardins-Crepeau et al	2016	Color-word inhibition	executive
Desjardins-Crepeau et al	2016	Color-word interference_color	speed
Desjardins-Crepeau et al	2016	Color-word interference_reading	speed
Desjardins-Crepeau et al	2016	Color-word task switching	executive
Desjardins-Crepeau et al	2016	Handgrip strength	strength
Desjardins-Crepeau et al	2016	Modified phys performance test	fitness
Desjardins-Crepeau et al	2016	RAVLT delayed	memory
Desjardins-Crepeau et al	2016	RAVLT immediate	memory
Desjardins-Crepeau et al	2016	RAVLT total	memory
Desjardins-Crepeau et al	2016	TMT A	speed
Desjardins-Crepeau et al	2016	TMT B	executive
Desjardins-Crepeau et al	2016	TUG	balance
Eggenberger et al	2015	Age concentration A	attention
Eggenberger et al	2015	Age concentration B	attention
Eggenberger et al	2015	Digit Span forward	memory
Eggenberger et al	2015	DSST	speed
Eggenberger et al	2015	Executive control task	executive
Eggenberger et al	2015	PALT	memory
Eggenberger et al	2015	Story recall	memory
Eggenberger et al	2015	TMT A	speed
Eggenberger et al	2015	TMT B	executive
Fabre et al	2002	Digit Span forward	memory
Fabre et al	2002	Logical memory - immediate	memory
Fabre et al	2002	Logical memory information	memory
Fabre et al	2002	Logical memory mental control	executive
Fabre et al	2002	Logical memory orientation	memory
Fabre et al	2002	Logical memory visual reprod	memory
Fabre et al	2002	O2 pulse	fitness
Fabre et al	2002	O2pulse max	fitness
Fabre et al	2002	PALT	memory
Fabre et al	2002	VO2	fitness
Fabre et al	2002	VOX2max	fitness
Fabre et al	2002	Wais memory quotient	memory
Gill et al	2016	Auditory verbal learning-learning	memory
Gill et al	2016	Autitory verbal learning-recall	memory
Gill et al	2016	DSST	speed
Gill et al	2016	Verbal fluency category	language
Gill et al	2016	Verbal fluency letter	language
Gschwind et al	2015	10-m walk-single task	fitness
Gschwind et al	2015	Attention network test-alert	attention
Gschwind et al	2015	Attention network test-conflict	attention
Gschwind et al	2015	Attention network test-orient	attention
Gschwind et al	2015	Attention network test-RT	attention

Gschwind et al	2015	Coordinated stability	balance
Gschwind et al	2015	Digit Span backward	executive
Gschwind et al	2015	DSST	speed
Gschwind et al	2015	Handgrip strength	strength
Gschwind et al	2015	Knee extension	strength
Gschwind et al	2015	Maximum balance range-antero posterior	balance
Gschwind et al	2015	Melbourne edge test	balance
Gschwind et al	2015	Propioception	balance
Gschwind et al	2015	Sensor-based chair stand test	strength
Gschwind et al	2015	Sensor-based full tandem stance	balance
Gschwind et al	2015	Sensor-based near tandem stance	balance
Gschwind et al	2015	Sensor-based semi tandem stance	balance
Gschwind et al	2015	SPPB	fitness
Gschwind et al	2015	Sway-area	balance
Gschwind et al	2015	TMT A	speed
Gschwind et al	2015	TMT B	executive
Gschwind et al	2015	TUG	balance
Gschwind et al	2015	Victoria Stroop-efficacy score	executive
Gschwind et al	2015	Victoria Stroop-intrusions	executive
Hiyamizu et al	2012	Chair stand test	strength
Hiyamizu et al	2012	Functional reach test	balance
Hiyamizu et al	2012	Stroop ACC	executive
Hiyamizu et al	2012	Sway - eyes closed	balance
Hiyamizu et al	2012	Sway - eyes open	balance
Hiyamizu et al	2012	TMT A	speed
Hiyamizu et al	2012	TMT B	executive
Hiyamizu et al	2012	TMT B-A	executive
Hiyamizu et al	2012	TUG	balance
Htut et al	2018	Five times sit to stand	strength
Htut et al	2018	Handgrip left	strength
Htut et al	2018	Handgrip right	strength
Htut et al	2018	MoCA	global
Htut et al	2018	TUG	balance
Jardim et al	2021	30-s chair stand	strength
Jardim et al	2021	6-m walk	fitness
Jardim et al	2021	CERARD word list - evocation	memory
Jardim et al	2021	CERARD word list - inm	memory
Jardim et al	2021	CERARD word list - recognition	memory
Jardim et al	2021	PALT - nº of patterns	memory
Jardim et al	2021	PALT - stages completed	memory
Jardim et al	2021	PALT - total	memory
Jardim et al	2021	Rapid visual processing	attention
Jardim et al	2021	TUG	balance
Jardim et al	2021	Walking m/s	fitness
Jehu et al	2017	Counting backward (TUGcog)	executive
Jehu et al	2017	TUG	balance
Joubert & Chainay	2019	Complex Span task - ACC	executive

Joubert & Chainay	2019	Complex Span task - RT	executive
Joubert & Chainay	2019	Flanker task - ACC	executive
Joubert & Chainay	2019	Flanker task - RT	executive
Joubert & Chainay	2019	Plus Minus task - ACC	executive
Joubert & Chainay	2019	Plus Minus task - RT	executive
Joubert & Chainay	2019	RAVLT- lexical	memory
Joubert & Chainay	2019	RAVLT-categories	memory
Joubert & Chainay	2019	TMT B-A	executive
Joubert & Chainay	2019	Updated Span task - ACC	executive
Joubert & Chainay	2019	Updated Span task - RT	executive
Kitazawa et al	2015	Dementia Assessment Scale	global
Kitazawa et al	2015	Touch-M - visuospatial	memory
Kitazawa et al	2015	TUG	balance
Laatar et al	2018	30-s chair stand test	strength
Laatar et al	2018	CoP x	balance
Laatar et al	2018	CoP y	balance
Laatar et al	2018	Functional reach test	balance
Laatar et al	2018	Gait speed	fitness
Laatar et al	2018	Simple reaction time	speed
Laatar et al	2018	TUG	balance
Legault et al	2011	1-back	memory
Legault et al	2011	2-back	executive
Legault et al	2011	Flanker task	executive
Legault et al	2011	HVLT delayed	memory
Legault et al	2011	HVLT immediate	memory
Legault et al	2011	HVLT suppl score	memory
Legault et al	2011	HVTL total	memory
Legault et al	2011	Self-ordered pointing task	executive
Legault et al	2011	Task switching	executive
Legault et al	2011	TMT B-A	executive
Linde & Alfermann	2014	d2 test of attention	attention
Linde & Alfermann	2014	DSST	speed
Linde & Alfermann	2014	Leistungs-Pruf-System 50+ Reasoning	speed
Linde & Alfermann	2014	Leistungs-Pruf-System 50+ Spatial relatio	speed
Linde & Alfermann	2014	TMT A	speed
Linde & Alfermann	2014	V02max	fitness
Linde & Alfermann	2014	Word list test	memory
Maillot & Hartley	2012	6-Min Walk test-distance	fitness
Maillot & Hartley	2012	6-Min Walk test-max HR	fitness
Maillot & Hartley	2012	6-Min Walk test-mean HR	fitness
Maillot & Hartley	2012	8-Foot Up-and Go test	fitness
Maillot & Hartley	2012	Arm curls	strength
Maillot & Hartley	2012	Back Scratch test – lower left	fitness
Maillot & Hartley	2012	Back Scratch test – lower right	fitness
Maillot & Hartley	2012	Back Scratch test – upper left	fitness
Maillot & Hartley	2012	Back Scratch test – upper right	fitness
Maillot & Hartley	2012	Cancellation test	speed

Maillot & Hartley	2012	Chair stand test	strength
Maillot & Hartley	2012	Directional Headings test	executive
Maillot & Hartley	2012	DSST	speed
Maillot & Hartley	2012	Letter Sets test	executive
Maillot & Hartley	2012	Matrix reasoning test	executive
Maillot & Hartley	2012	Mental rotation test	executive
Maillot & Hartley	2012	Number comparison test	speed
Maillot & Hartley	2012	Reaction time test – choice	speed
Maillot & Hartley	2012	Reaction time test – simple	speed
Maillot & Hartley	2012	Spatial Span test	executive
Maillot & Hartley	2012	Spatial Span test - backward	executive
Maillot & Hartley	2012	Stroop incongruent	executive
Maillot & Hartley	2012	Stroop switching	executive
Maillot & Hartley	2012	TMT B-A	executive
Marmeleira et al.	2009	Dual-task movement time	attention
Marmeleira et al.	2009	Dual-task reaction time	attention
Marmeleira et al.	2009	Dual-task response time	attention
Marmeleira et al.	2009	Foot tap test (Lower limb mobility)	fitness
Marmeleira et al.	2009	Functional reach test	balance
Marmeleira et al.	2009	Self-only in motion. Absolute errors	attention
Marmeleira et al.	2009	Self-only in motion. Constant errors	attention
Marmeleira et al.	2009	Self-only in motion. Variable errors	attention
Marmeleira et al.	2009	Single-task. Movement time	speed
Marmeleira et al.	2009	Single-task. Reaction time	speed
Marmeleira et al.	2009	Single-task. Response time	speed
Marmeleira et al.	2009	Stroop - incongruent	executive
Marmeleira et al.	2009	Stroop - interference	executive
Marmeleira et al.	2009	Target-only in motion. Absolute errors	attention
Marmeleira et al.	2009	Target-only in motion. Constant errors	attention
Marmeleira et al.	2009	Target-only in motion. Variable errors	attention
Marmeleira et al.	2009	Three-choice reaction time	speed
Marmeleira et al.	2009	TMT B errors	executive
Marmeleira et al.	2009	TMT B s	executive
Marmeleira et al.	2009	TUG	balance
Marmeleira et al.	2009	Two-choice reaction time	speed
Marmeleira et al.	2009	Useful Field of View - divided att	attention
Marmeleira et al.	2009	Useful Field of View - selective att	attention
Marmeleira et al.	2009	Useful Field of View - speed	speed
McDaniel et al	2014	Cooking Breakfast Task- Ideal Performance	memory
McDaniel et al	2014	Cooking Breakfast Task- Number of Table S	memory
McDaniel et al	2014	Cooking Breakfast Task- Stopping Time Ran	memory
McDaniel et al	2014	Memory for Health Information Task- Corre	memory
McDaniel et al	2014	Memory for Health Information Task- FAs t	memory
McDaniel et al	2014	Memory for Health Information Task- Sourc	memory
McDaniel et al	2014	Virtual Week Task - irregular	attention
McDaniel et al	2014	Virtual Week Task - regular	attention
McDaniel et al	2014	Virtual Week Task – time based	attention

McDaniel et al	2014	VO2peak	fitness
Morita et al	2018	Maximal step length	fitness
Morita et al	2018	Modified Mini-Mental State (3MS)	global
Morita et al	2018	Quad. Muscle strength	strength
Morita et al	2018	Single-leg standing	balance
Morita et al	2018	TUG	balance
Ng et al	2017	RBANS - attention	attention
Ng et al	2017	RBANS - language	language
Ng et al	2017	RBANS - total	composite
Ng et al	2017	RBANS - visuospatial	executive
Ng et al	2017	RBANS – delayed	memory
Ng et al	2017	RBANS – immediate	memory
Ngandu et al	2015	Neuropsychological test battery - Executive Functions	executive
Ngandu et al	2015	Neuropsychological test battery - Memory	memory
Ngandu et al	2015	Neuropsychological test battery - Processing speed	speed
Ngandu et al	2015	Neuropsychological test battery – Memory short version	memory
Ngandu et al	2015	Neuropsychological test battery (NTB) – total	composite
Nilsson et al	2020	Episodic memory spatial + verbal	memory
Nilsson et al	2020	ETS kit verbal inference + BIS analogies + Syllogisms	language
Nilsson et al	2020	n-back + Running span trained	speed
Nilsson et al	2020	n-back + Running span untrained	speed
Nilsson et al	2020	Numerical and spatial updating	speed
Nilsson et al	2020	Perceptual matching 1+ 2	speed
Nilsson et al	2020	Raven's Progressive Matrices	executive
Nilsson et al	2020	Task switching 1 + 2	speed
Nishiguchi et al	2015	0-back - face-ACC	speed
Nishiguchi et al	2015	0-back - face-ms	speed
Nishiguchi et al	2015	0-back - face+location ACC	speed
Nishiguchi et al	2015	0-back - face+location-ms	speed
Nishiguchi et al	2015	0-back - location-ACC	speed
Nishiguchi et al	2015	0-back - location-ms	speed
Nishiguchi et al	2015	1-back face+location-ms	executive
Nishiguchi et al	2015	1-back - face-ms	executive
Nishiguchi et al	2015	1-back - face+location-ms	executive
Nishiguchi et al	2015	1-back - location-ACC	executive
Nishiguchi et al	2015	1-back - location-ms	executive
Nishiguchi et al	2015	10-m walk test	fitness
Nishiguchi et al	2015	Chair stand test	strength
Nishiguchi et al	2015	Daily steps	fitness
Nishiguchi et al	2015	Logical memory - delayed	memory
Nishiguchi et al	2015	Logical memory - immediate	memory
Nishiguchi et al	2015	MMSE	global
Nishiguchi et al	2015	TMT B-A	executive
Nishiguchi et al	2015	TUG	balance
Nocera et al	2020	Digit Span backward	executive
Nocera et al	2020	Digit Span forward	memory

Nocera et al	2020	Letter fluency	language
Nocera et al	2020	n-back-ACC	executive
Nocera et al	2020	n-back-ms	executive
Nocera et al	2020	Semantic fluency	language
Nocera et al	2020	Single gait	fitness
Nocera et al	2020	SPPB	fitness
Nocera et al	2020	Stroop	executive
Nocera et al	2020	TMT A	speed
Nocera et al	2020	TMT B	executive
Nocera et al	2020	VO2	fitness
Norouzi et al	2019	Berg Balance Scale	balance
Norouzi et al	2019	n-back	executive
Oswald et al	2006	Composite z score - cognitive	composite
Phirom et al	2020	MoCA	global
Phirom et al	2020	Physiological Profile Assessment – knee extension strength	strength
Phirom et al	2020	Physiological Profile Assessment – Sway	balance
Phirom et al	2020	TUG – single task	balance
Pieramico et al	2012	Babcock Story – Delayed Recall	memory
Pieramico et al	2012	Babcock Story – Immediate Recall	memory
Pieramico et al	2012	Babcock Story Recall Test	memory
Pieramico et al	2012	Frontal Assessment Battery	global
Pieramico et al	2012	MMSE	global
Pieramico et al	2012	Phonological Fluency test	language
Pieramico et al	2012	TMT A	speed
Pieramico et al	2012	TMT B	executive
Pieramico et al	2012	TMT B-A	executive
Rahe et al (a)	2015	30 s Chair stand	strength
Rahe et al (a)	2015	6 minute walk test/2Min step test	fitness
Rahe et al (a)	2015	8 foot up and go test	fitness
Rahe et al (a)	2015	Arm curl	strength
Rahe et al (a)	2015	Brief Test of Attention	attention
Rahe et al (a)	2015	Chair sit and reach test	fitness
Rahe et al (a)	2015	Complex Figure Test- memory	memory
Rahe et al (a)	2015	DemTect - composite	composite
Rahe et al (a)	2015	DemTect subtest supermarket/animal	language
Rahe et al (a)	2015	DemTect-delayed recall	memory
Rahe et al (a)	2015	DemTect-immediate recall	memory
Rahe et al (a)	2015	Digit Span backward	executive
Rahe et al (a)	2015	Overal fitness	fitness
Rahe et al (a)	2015	Regensburger Wort Flüssigkeits-Test – Fluidez verbal	language
Rahe et al (a)	2015	Stroop	executive
Rahe et al (b)	2015	Brief Test of Attention	attention
Rahe et al (b)	2015	Complex Figure Test- memory	global
Rahe et al (b)	2015	COWAT	language
Rahe et al (b)	2015	TMT B/A	executive
Raichlen et al	2020	serially subtract 7's beginning at 500	executive

Raichlen et al	2020	Stride duration	balance
Raichlen et al	2020	Stride duration variability	balance
Raichlen et al	2020	Stride length	balance
Raichlen et al	2020	Stride length variability	balance
Raichlen et al	2020	Stride velocity	balance
Raichlen et al	2020	Stride velocity variability	balance
Romera-Liebana et al	2018	Abstraction of word pairs	language
Romera-Liebana et al	2018	Animal naming test	language
Romera-Liebana et al	2018	Designation of images	language
Romera-Liebana et al	2018	Designation of names	language
Romera-Liebana et al	2018	Evocation of words	language
Romera-Liebana et al	2018	Functional reach test	fitness
Romera-Liebana et al	2018	Handgrip	strength
Romera-Liebana et al	2018	SPPB	fitness
Romera-Liebana et al	2018	Unipodal station	fitness
Romera-Liebana et al	2018	Verbal memory delayed	memory
Romera-Liebana et al	2018	Verbal memory immediate	memory
Salazar et al	2014	Cadence	balance
Salazar et al	2014	Double support	balance
Salazar et al	2014	Step width	balance
Salazar et al	2014	Stride length	balance
Salazar et al	2014	Substracting digits backwards	executive
Salazar et al	2014	Swing	balance
Salazar et al	2014	Walk speed	fitness
Schoene et al (a)	2013	Alternate Step test	balance
Schoene et al (a)	2013	Chair stand test	strength
Schoene et al (a)	2013	Choice stepping reaction time. Movement time.	speed
Schoene et al (a)	2013	Choice stepping reaction time. Reaction time.	speed
Schoene et al (a)	2013	Choice stepping reaction time. Total response time.	speed
Schoene et al (a)	2013	Physiological Profile Assessment (PPA). anteroposterior	balance
Schoene et al (a)	2013	Physiological Profile Assessment (PPA). central	balance
Schoene et al (a)	2013	Physiological Profile Assessment (PPA). medio-lateral	balance
Schoene et al (a)	2013	Physiological Profile Assessment (PPA). Global	balance
Schoene et al (a)	2013	Physiological Profile Assessment (PPA). Lower extremity strength	strength
Schoene et al (a)	2013	Physiological Profile Assessment (PPA). Propioception of lower extremities	balance
Schoene et al (a)	2013	Step inhibition test - s	executive
Schoene et al (a)	2013	Step inhibition test errors	executive
Schoene et al (a)	2013	Step inhibition test time/trials	executive
Schoene et al (a)	2013	TMT A	speed
Schoene et al (a)	2013	TUG	balance
Schoene et al (b)	2015	Attentional network test - alert	attention
Schoene et al (b)	2015	Attentional network test - executive	executive
Schoene et al (b)	2015	Attentional network test - orientation	attention
Schoene et al (b)	2015	Choice stepping movement time test	speed
Schoene et al (b)	2015	Choice stepping reaction time test	speed

Schoene et al (b)	2015	Digit letter maximum	speed
Schoene et al (b)	2015	Digit letter mean	speed
Schoene et al (b)	2015	Digit letter minimum	speed
Schoene et al (b)	2015	Digit Span backward	executive
Schoene et al (b)	2015	Hand reaction time test	speed
Schoene et al (b)	2015	Mental rotation - errors	executive
Schoene et al (b)	2015	Mental rotation - TR	executive
Schoene et al (b)	2015	Stroop - errors	executive
Schoene et al (b)	2015	Stroop - TR	executive
Schoene et al (b)	2015	Stroop Stepping Test - TR	executive
Schoene et al (b)	2015	Stroop Stepping Test -errors	executive
Schoene et al (b)	2015	TMT A	speed
Schoene et al (b)	2015	TMT B	executive
Schoene et al (b)	2015	TMT B/A	executive
Shah et al	2014	1-back	memory
Shah et al	2014	Borg's scale	balance
Shah et al	2014	COWAT	language
Shah et al	2014	Detection (DET)	speed
Shah et al	2014	Groton Maze learning	memory
Shah et al	2014	Immediate Recall	memory
Shah et al	2014	Incremental Shuttle Walk test	fitness
Shah et al	2014	long term delayed recall	memory
Shah et al	2014	short term delayed recall	memory
Shah et al	2014	Sum of Strength (kgs.)	strength
Shah et al	2014	Visual Memory - index score	memory
Shatil et al	2013	CogniFit avoiding distractors	executive
Shatil et al	2013	CogniFit divided attention	attention
Shatil et al	2013	CogniFit global visual memory	memory
Shatil et al	2013	CogniFit inhibition	executive
Shatil et al	2013	CogniFit naming	language
Shatil et al	2013	CogniFit planning	executive
Shatil et al	2013	CogniFit processing speed	speed
Shatil et al	2013	CogniFit self-awareness	executive
Shatil et al	2013	CogniFit shifting	executive
Shatil et al	2013	CogniFit time estimation	executive
Shatil et al	2013	CogniFit visual scanning	attention
Shatil et al	2013	CogniFit working memory	executive
Takeuchi et al	2020	0-back	memory
Takeuchi et al	2020	2-back	executive
Takeuchi et al	2020	Digit cancellation task	attention
Takeuchi et al	2020	Digit span	executive
Takeuchi et al	2020	Frontal lobe and executive function	executive
Takeuchi et al	2020	Logical memory	memory
Takeuchi et al	2020	Raven's Progressive Matrices	executive
Takeuchi et al	2020	Semantic fluency	language
Takeuchi et al	2020	Symbol search	speed
Teixeira et al	2013	Digit Span backward	executive

Teixeira et al	2013	Digit Span forward	memory
Teixeira et al	2013	MMSE	global
Teixeira et al	2013	Modified Card Sorting Test - errors	executive
Teixeira et al	2013	Modified Card Sorting Test - errors adjusted for age	executive
Teixeira et al	2013	Toulouse-Pierón Concentrated Attention Test - hits	attention
Teixeira et al	2013	Toulouse-Pierón Concentrated Attention Test - TR	attention
Theill et al.	2013	Continuous Performance task	attention
Theill et al.	2013	DSST	speed
Theill et al.	2013	Dual task (WM+gait) - errors	executive
Theill et al.	2013	Dual task (WM+gait) - hits	executive
Theill et al.	2013	Executive control task	executive
Theill et al.	2013	Gait variability – single task	fitness
Theill et al.	2013	Gait velocity – single task	fitness
Theill et al.	2013	Operation span test	memory
Theill et al.	2013	PALT	memory
Theill et al.	2013	Raven's Progressive Matrices	executive
Van het Reve & de Bruin	2014	TMT A	speed
Van het Reve & de Bruin	2014	TMT B	executive
Van het Reve & de Bruin	2014	Vienna Test System – divided attention, lower channel	attention
Van het Reve & de Bruin	2014	Vienna Test System – divided attention, upper channel	attention
Wollesen et al (a)	2017	Gait-line left	balance
Wollesen et al (a)	2017	Gait-line right	balance
Wollesen et al (a)	2017	SPPB	balance
Wollesen et al (a)	2017	Step length left	balance
Wollesen et al (a)	2017	Step length right	balance
Wollesen et al (a)	2017	Step width	balance
Wollesen et al (a)	2017	Stroop	executive
Wollesen et al (a)	2017	Stroop dual-task (during walking)	executive
Wollesen et al (b)	2017	Gait-line left	balance
Wollesen et al (b)	2017	Gait-line right	balance
Wollesen et al (b)	2017	SPPB	balance
Wollesen et al (b)	2017	Step length left	balance
Wollesen et al (b)	2017	Step length right	balance
Wollesen et al (b)	2017	Step width	balance
Wollesen et al (b)	2017	Stroop	executive
Wollesen et al (b)	2017	Stroop dual-task (during walking)	executive
Wongcharoen et al	2017	Counting backwards	speed
Wongcharoen et al	2017	Step width	balance
Wongcharoen et al	2017	Stride length	balance
Wongcharoen et al	2017	Verbal fluency test	language
Wongcharoen et al	2017	XcoM-BoS - Narrow walk distance	balance
Wongcharoen et al	2017	XcoM-BoS - Narrow walk speed	balance
Yokoyama et al	2015	Maximal step length	fitness
Yokoyama et al	2015	Modified Mini-Mental State (3MS)	global
Yokoyama et al	2015	Muscle strength - legs	strength
Yokoyama et al	2015	Muscle strength – cuadr.	strength
Yokoyama et al	2015	Single leg standing	balance

Yokoyama et al	2015	TMT A	speed
Yokoyama et al	2015	TUG	balance
You et al	2009	Gait stability - AP COP	balance
You et al	2009	Gait stability - ML COP	balance
You et al	2009	Gait velocity	fitness
You et al	2009	Memory recall	memory
Yu et al (dual-cognitive)	2021	Frontal Assessment Battery	global
Yu et al (dual-cognitive)	2021	Hong Kong List Learning Test-Delay Recall Trial	memory
Yu et al (dual-cognitive)	2021	Hong Kong List Learning Test-Total Learning	memory
Yu et al (dual-cognitive)	2021	Rapid Cognitive Screen	global
Yu et al (multicognitive)	2021	Frontal Assessment Battery	global
Yu et al (multicognitive)	2021	Hong Kong List Learning Test-Delay Recall Trial	memory
Yu et al (multicognitive)	2021	Hong Kong List Learning Test-Total Learning	memory
Yu et al (multicognitive)	2021	Rapid Cognitive Screen	global

Table S3. Quality assessment of the reviewed articles using the Checklist for Assessing the Quality of Quantitative Studies (Kmet, Lee, and Cook (2004).

Criteria	1	2	3	4	5	6	7	8	9	10	11	12	13	14	Quality score	Groups
Fabre et al. (2002)	Y	Y	Y	Y	Y	N	N	Y	N	Y	Y	Y	Y	Y	22	Combined; single physical; single cognitive; active control
Oswald et al. (2006)	Y	Y	Y	Y	N	N	N	Y	P	Y	Y	P	Y	Y	20	Combined; single physical, single cognitive, passive control; psychoeducation (not incl.); psychoeducation + physical (not incl.)
Marmeleira et al. (2009)	Y	P	Y	Y	P	N	N	Y	N	Y	Y	P	Y	Y	19	Combined; passive control
You et al. (2009)	Y	Y	Y	Y	Y	N	N	Y	N	Y	Y	P	Y	Y	21	Combined; physical + cognitive control
Legault et al. (2011)	Y	Y	Y	Y	Y	N	Y	Y	N	Y	Y	P	Y	Y	23	Combined; single physical; single cognitive; active control
Anderson-Hanley et al. (2012).	Y	Y	Y	Y	Y	N	N	Y	Y	Y	Y	P	Y	Y	23	Combined; single physical
Hiyamizu et al. (2012).	Y	Y	Y	Y	Y	Y	N	Y	N	Y	Y	P	Y	Y	23	Combined; single physical
Maillot et al. (2012)	Y	P	Y	Y	P	N	N	Y	N	Y	Y	Y	Y	Y	20	Combined; passive control
Pieramico et al. (2012)	Y	y	Y	Y	P	N	N	Y	N	Y	Y	P	Y	Y	20	Combined; passive control
Schoene et al. (2013)	Y	Y	y	y	P	Y	N	Y	P	Y	Y	Y	Y	Y	24	Combined, passive control
Shatil (2013)	Y	y	y	Y	Y	N	N	Y	P	Y	Y	P	Y	Y	22	Combined; single physical, single cognitive, passive control
Teixeira et al., (2013)	Y	Y	Y	Y	N	N	N	Y	N	Y	Y	N	Y	Y	18	Combined; passive control
Theill et al. (2013)	Y	Y	N	Y	N	N	N	Y	N	Y	Y	P	Y	Y	17	Combined, single cognitive, passive control
Linde & Alfermann (2014)	Y	Y	Y	Y	Y	Y	N	Y	N	Y	Y	P	Y	Y	23	Combined, single physical, single cognitive, passive control
McDaniel et al. (2014)	Y	Y	Y	Y	P	N	N	Y	N	Y	Y	Y	Y	Y	21	Combined, physical + cognitive control, cognitive + physical control, physical control + cognitive control
Salazar et al. (2014)	Y	Y	Y	Y	N	N	N	Y	Y	Y	Y	N	Y	Y	20	Combined, passive control
Shah et al. (2014)	Y	Y	Y	Y	N	N	N	Y	P	Y	Y	P	y	Y	20	Combined, single physical, single cognitive, passive control
Van Het Reve & de Bruin (2014)	Y	Y	Y	Y	Y	N	N	Y	Y	Y	Y	Y	Y	Y	24	Combined, single physical
Bamidis et al. (2015)	Y	Y	Y	Y	N	N	N	Y	Y	Y	Y	Y	Y	Y	22	Combined, passive control
Eggenberger et al. (2015)	Y	N	Y	Y	Y	N	Y	Y	Y	Y	Y	P	Y	Y	23	Combined, single physical
Gschwind et al. (2015)	Y	Y	Y	Y	Y	Y	Y	Y	Y	Y	Y	Y	Y	Y	28	Combined, passive control
Kitazawa et al. (2015)	Y	Y	Y	P	Y	N	N	Y	N	Y	Y	P	Y	Y	18	Combined, passive control
Ngandu et al. (2015)	Y	y	y	y	Y	y	Y	y	Y	Y	y	y	y	Y	28	Combined, active control
Nishiguchi et al (2015)	Y	Y	Y	Y	Y	Y	N	Y	Y	Y	Y	Y	Y	Y	26	Combined, passive control
Rahe et al. (2015a)	Y	P	Y	Y	N	N	N	Y	N	Y	Y	N	Y	Y	17	Combined, single cognitive

Rahe et al. (2015b)	Y	P	Y	Y	N	N	N	Y	N	Y	Y	N	Y	Y	17	Combined, single cognitive
Schoene et al. (2015)	Y	Y	y	y	Y	Y	N	Y	Y	Y	Y	Y	y	Y	26	Combined, passive control
Yokoyama et al. (2015)	Y	P	Y	Y	Y	N	Y	Y	N	Y	Y	P	Y	Y	22	Combined, single physical
Desjardins-Crépeau et al. (2016)	Y	Y	Y	Y	Y	Y	N	Y	N	Y	Y	Y	Y	Y	24	Combined, single physical + cognitive control, single cognitive + physical control, physical control + cognitive control
Gill et al. (2016)	Y	N	Y	Y	Y	Y	N	Y	Y	Y	Y	P	Y	Y	23	Combined, single physical
Andrieu et al. (2017)	Y	Y	Y	Y	Y	N	P	Y	Y	Y	Y	Y	Y	Y	25	Combined, passive control
Barban et al. (2017)	Y	Y	Y	Y	Y	Y	P	Y	Y	Y	Y	Y	Y	Y	27	Combined, single physical, single cognitive, control
Jehu et al. (2017)	Y	Y	Y	Y	P	N	N	Y	N	Y	Y	P	Y	Y	20	Combined, single physical, passive control
Ng et al. (2018)	Y	P	Y	Y	Y	Y	N	Y	Y	Y	Y	N	Y	Y	23	Combined, single physical, single cognitive, active control
Wollesen et al. (2017)	Y	Y	Y	Y	Y	N	N	Y	Y	Y	Y	Y	Y	Y	23	Combined with and without concern of falling, passive control
Wongcharoen et al. (2017)	Y	Y	Y	Y	Y	N	N	Y	N	Y	Y	P	Y	Y	20	Combined, single physical, single cognitive
Htut et al. (2018)	Y	Y	Y	Y	Y	Y	N	P	Y	Y	Y	P	Y	Y	24	Combined, passive control
Laatar et al. (2018)	Y	P	Y	Y	Y	N	N	Y	N	Y	Y	P	Y	Y	2O	Combined, single physical
Morita et al. (2018)	Y	P	Y	P	N	N	N	Y	N	Y	Y	N	Y	Y	16	Combined, passive control
Romera-Liebana et al. (2018)	Y	Y	Y	Y	P	Y	N	Y	Y	Y	Y	P	Y	Y	24	Combined, passive control
Joubert & Chainay, (2019)	Y	Y	Y	Y	P	N	N	Y	N	Y	Y	P	Y	Y	20	Combined, single cognitive, passive control
Norouzi et al. (2019)	Y	Y	Y	Y	P	N	N	Y	N	Y	Y	P	Y	Y	20	Combined, passive control, single physical (not incl.: nonequivalent exercise intervention)
Adcock et al. (2020)	Y	Y	Y	Y	Y	N	N	Y	N	Y	Y	N	Y	Y	20	Combined, passive control
Nilsson et al. (2020)	Y	Y	Y	Y	Y	N	Y	Y	Y	Y	Y	Y	Y	Y	26	Combined, single physical, single cognitive
Nocera et al. (2020)	Y	Y	Y	Y	P	N	N	Y	N	Y	Y	N	Y	Y	19	Combined, single physical, single cognitive + physical control
Phirom et al. (2020)	Y	Y	Y	Y	N	N	N	Y	N	Y	Y	Y	Y	Y	20	Combined, passive control
Raichlen et al. (2020)	Y	Y	Y	Y	Y	N	N	N	N	Y	Y	Y	Y	Y	20	Combined, single physical, single cognitive, active control
Takeuchi et al. (2020)	Y	Y	Y	Y	Y	N	N	Y	Y	Y	Y	Y	Y	Y	22	Combined, single physical, single cognitive
Jardim et al. (2021)	Y	Y	Y	Y	Y	Y	N	Y	Y	Y	Y	P	Y	Y	25	Combined, passive control
Yu et al. (2021)	Y	Y	Y	Y	Y	N	N	Y	Y	Y	Y	Y	Y	Y	22	Combined, single physical + cognitive control

Note. Y: Yes; P: Partial; N: No; N/A: Not applicable. 1: Question / objective sufficiently described?; 2: Study design evident and appropriate?; 3: Method of subject/comparison group selection or source of information/input variables described and appropriate?; 4: Subject (and comparison group, if applicable) characteristics sufficiently described?; 5: If interventional and random allocation was possible, was it described?; 6: If interventional and blinding of investigators was possible, was it reported?; 7: If interventional and blinding of subjects was possible, was it reported?; 8: Outcome and (if applicable) exposure measure(s) well defined and robust to measurement / misclassification bias? Means of assessment reported?; 9: Sample size appropriate?; 10: Analytic methods described/justified and appropriate?; 11: Some estimate of variance is reported for the main results?; 12: Controlled for confounding; 13: Results reported in sufficient detail?; 14: Conclusions supported by the results? Yes = 2 points; Partial = 1 point; No = 0 points; N/A = not applicable

Table S4. Results of the influential case analysis

Authors	ES	v	Function	Comparison	Rstudent	dffits	cook.d	cov.r	tau2.del	QE.del	hat	weight	dfbs inf
**Pre-post outcomes**													
Oswald et al, 2016	1.0096	.057	composite	comb-control	2.1472	0.1667	0.0272	0.9824	0.0448	1204.5378	0.0025	0.2481	0.1666
Anderson-Hanley et al, 2012	-1.5200	.084	memory	comb-physical	3.5554	0.2944	0.0838	0.9593	0.0311	1264.8094	0.0017	0.1698	0.2957
Anderson-Hanley et al, 2012	1.2280	.077	executive	comb-physical	2.7594	0.2100	0.0431	0.9749	0.0326	1271.4589	0.0018	0.1804	0.2105
Maillot & Hartley, 2012	2.0486	.218	speed	comb-control	3.4601	0.1652	0.0271	0.9808	0.0332	1268.7936	0.0008	0.0800	0.1663
Maillot & Hartley, 2012	2.1280	.224	executive	comb-control	3.5739	0.1700	0.0287	0.9800	0.0332	1267.9221	0.0008	0.0780	0.1711
Schoene et al (a), 2013	3.4110	.338	executive	comb-control	5.0884	0.2171	0.0468	0.9712	0.0324	1254.1454	0.0005	0.0542	0.2196
Nishiguchi et al, 2015	-8.0860	.834	speed	comb-control	8.3542	0.2412	0.0580	0.9662	0.0319	1210.0463	0.0002	0.0233	0.2450
Nishiguchi et al, 2015	-7.6470	.754	speed	comb-control	8.2711	0.2540	0.0642	0.9639	0.0317	1211.1985	0.0003	0.0256	0.2582
Schoene et al (b), 2015	9.9500	.696	executive	comb-control	11.3233	0.4170	0.1724	0.9324	0.0288	1148.7103	0.0003	0.0277	0.4301
Yokoyama et al, 2015	2.9840	.380	global	comb-physical	4.1520	0.1524	0.0231	0.9824	0.0334	1263.9084	0.0005	0.0487	0.1535
Wollesen et al (b), 2017	1.6040	.161	executive	comb-control	2.9265	0.1553	0.0239	0.9830	0.0334	1272.2366	0.0010	0.1034	0.1560
Norouzi et al, 2019	5.5560	.534	executive	comb-control	6.9692	0.2514	0.0628	0.9646	0.0318	1231.0176	0.0004	0.0355	0.2553
Nocera et al, 2010	-2.9600	.376	executive	comb-cognitivo	4.1320	0.1523	0.0231	0.9824	0.0334	1264.0738	0.0005	0.0492	0.1534
Jardim et al, 2021	1.4150	.062	memory	comb-control	3.6317	0.3573	0.1211	0.9485	0.0301	1262.3100	0.0021	0.2082	0.3577
Jardim et al, 2021	2.0780	.089	memory	comb-control	5.1789	0.5077	0.2418	0.9188	0.0274	1246.7427	0.0016	0.1630	0.5134
Jardim et al, 2021	2.5250	.104	memory	comb-control	6.1438	0.5996	0.3355	0.9006	0.0258	1234.6876	0.0015	0.1452	0.6108
Jardim et al, 2021	2.6080	.107	memory	comb-control	6.3031	0.6119	0.3493	0.8982	0.0256	1232.5822	0.0014	0.1419	0.6241
Jardim et al, 2021	2.7550	.113	memory	comb-control	6.5727	0.6306	0.3712	0.8943	0.0252	1228.9394	0.0014	0.1364	0.6447
Jardim et al, 2021	3.1890	.132	memory	comb-control	7.2711	0.6641	0.4134	0.8872	0.0246	1219.0715	0.0012	0.1208	0.6830
Jardim et al, 2021	3.4670	.146	balance	comb-control	5.5576	0.6506	0.3528	0.8106	0.1557	741.3390	0.0044	0.4406	0.6612
Jardim et al, 2021	4.5690	.211	fitness	comb-control	7.0074	0.7842	0.5068	0.7609	0.1408	724.4046	0.0037	0.3731	0.8176
Jardim et al, 2021	4.9500	.238	fitness	comb-control	7.3955	0.8000	0.5312	0.7537	0.1386	720.0702	0.0035	0.3511	0.8408
Jardim et al, 2021	5.3960	.272	strength	comb-control	7.7886	0.8047	0.5438	0.7500	0.1376	715.7461	0.0033	0.3266	0.8527
Nishiguchi et al, 2015	2.015	.131	fitness	comb-control	2.6774	0.2435	0.0566	0.9551	0.2228	738.3822	0.0049	0.4949	0.2440
Andrieu et al, 2017	2.3437	6.67	executive	comb-control	identified based on visual inspection of the funnel plot								
**Pre-follow up outcomes**													
Norouzi et al, 2019	2,8620	,216	executive	comb-control	identified based on visual inspection of the funnel plot								

Table S5. Results of the continuous and categorical moderator analyses by cognitive functions.

	Mean difference in ES [95% CI] by moderators in cognitive outcomes						
	**Executive functions**	**Memory**	**Attention**	**Language**	**Speed**	**Global**	**Composite**
**Continuous moderators**							
Quality	-0.014 [-0.042, 0.013]	-0.022 [-0.014, 0.058]	-0.028 [-0.067, 0.012]	-0.006 [-0.057, 0.056]	-0.014 [-0.068, 0.04]	-0.029 [-0.142, 0.084]	-0.075 [-0.211, 0.061]
Year	-0.013 [-0.037, 0.011]	-0.009 [-0.033, 0.016]	0.003 [-0.044, 0.049]	0.012 [-0.037, 0.058]	-0.044 [-0.094, 0.007]	0.039 [-0.041, 0.119]	-0.053 [-0.108, -0.001]
N	-0.006 [-0.001, 0.000]	-0.000 [-0.001, 0.000]	-0.003 [-0.004, -0.001] ***	-0.000 [-0.001, 0.001]	-0.000 [-0.001, 0.001]	-0.001 [-0.002, 0.000]	-0.000 [-0.002, 0.001]
Age mean	-0.003 [-0.022, 0.016]	-0.001 [-0.022, 0.02]	-0.014 [-0.038, 0.01]	0.015 [-0.018, 0.047]	0.009 [-0.026, 0.044]	-0.025 [-0.089, 0.038]	0.043 [0.025, 0.011]
Age SD	0.013 [-0.034, 0.061]	0.043 [-0.009, 0.095]	-0.019 [-0.103, 0.064]	0.02 [-0.04, 0.08]	-0.04 [-0.123, 0.042]	-0.112 [-0.437, 0.214]	-0.001 [-0.263, 0.266]
Nº sessions	-0.000 [-0.003, 0.002]	-0.000 [-0.002, 0.000]	0.001 [-0.004, 0.006]	0.000 [-0.000, 0.001]	0.000 [-0.001, 0.002]	-0.001 [-0.002, 0.000]	-0.001 [-0.002, 0.001]
Training/wks	-0.002 [-0.014, 0.011]	-0.003 [-0.01, 0.003]	-0.004 [-0.03, 0.022]	0.001 [-0.003, 0.006]	0.003 [-0.003, 0.009]	-0.002 [-0.009, 0.005]	-0.003 [-0.013, 0.007]
Minutes/week	-0.001 [-0.002, 0.000]	-0.000 [-0.001, 0.001]	-0.000 [-0.002, 0.001]	-0.001 [-0.002, 0.000]	-0.001 [-0.002, 0.000]	-0.001 [-0.005, 0.001]	-0.002 [-0.006, 0.001]
Min. cogn./week	-0.000 [-0.002, 0.001]	0.000 [-0.001, 0.002]	0.002 [-0.000, 0.005]	-0.001 [-0.003, 0.000]	-0.001 [-0.003, 0.001]	-0.001 [-0.006, 0.004]	-0.002 [-0.008, 0.007]
Min. phys/week	0.000 [-0.002, 0.002]	-0.000 [-0.002, 0.02]	-0.000 [-0.001, 0.003]	-0.001 [-0.003, 0.001]	-0.001 [-0.004, 0.002]	-0.002[-0.012, 0.008]	-0.004 [-0.008, 0.000]
**Combinatory mode**							
Simultaneous	0.208 [0.098, 0.318] ***	0.154 [-0.012, 0.321]	0.144 [0.017, 0.271] *	0.06 [-0.18, 0.302]	0.293 [0.1, 0.486] **	0.56 [-0.124, 0.996] *	NA
Sequential	0.157 [-0.034, 0.348]	0.074 [-0.088, 0.237]	0.286 [0.071, 0.5] *	0.023 [-0.229, 0.275]	-0.007 [-0.276, 0.262]	0.156 [-0.592, 0.904]	0.373 [-0.001, 0.748]
Separate days	0.175 [0.001, 0.349] *	0.16 [-0.003, 0.324]	-0.139 [-0.344, 0.067]	0.176 [-0.037, 0.388]	0.138[-0.242, 0.519]	0.161 [-0.549, 0.872]	0.003 [-0.354, 0.359]
**Aerobic vs non-aerobic**							
Aerobic	0.2 [0.087, 0.313] ***	0.108 [-0.016, 0.233]	0.279 [0.097, 0.461] **	0.088 [-0.173, 0.348]	0.175 [-0.058, 0.407]	-0.049 [-0.627, 0.53]	0.55 [-0.179, 1.279]
Non-aerobic	0.138 [0.053, 0.223] **	0.08 [-0.066, 0.226]	0.032 [-0.094, 0.157]	0.078 [-0.094, 0.25]	0.202 [0.034, 0.37] *	0.508 [0.149, 0.868] **	0.113 [-0.24, 0.465]
**Cognitive training type**							
Interactive[a]	0.322 [0.179, 0.465] ***	0.258 [-0.005, 0.521]	0.158 [-0.095, 0.411]	NA	0.494 [0.257, 0.731] ***	0.56 [0.124, 0.996] *	NA
Computer	0.131 [0.025, 0.227] *	0.059 [-0.07, 0.19]	0.069 [-0.139, 0.277]	0.047 [-0.177, 0.271]	0.042 [-0.152, 0.235]	0.046 [-0.899, 0.992]	NA
Multicomponent[b]	0.137 [-0.037, 0.31]	0.196 [0.033, 0.358] *	0.14 [-0.081, 0.362]	0.228 [0.036, 0.421] *	0.312 [0.009, 0.614] *	0.206 [-0.409, 0.821]	0.111 [-0.218, 0.447]
**Setting**							
Group	0.162 [0.068, 0.256] ***	0.182 [0.058, 0.305] **	0.189 [0.047, 0.331] *	0.207 [0.012, 0.402] *	0.241 [0.038, 0.443] *	0.482 [0.027, 0.938] *	0.28 [-1.033, 1.593]
Individual	0.151 [0.022, 0.279] *	0.111 [-0.117, 0.339]	0.032 [-0.181, 0.245]	0.086 [-0.399, 0.227]	0.08 [-0.18, 0.34]	0.42 [-0.378, 1.219]	NA
Mixed	0.195 [-0.119, 0.51]	0.198 [-0.023, 0.42]	NA	0.333 [0.028, 0.638] *	0.348 [0.015, 0.68] *	0.162 [-0.592, 0.016]	NA

Note. ES = Hedges' g; CI = confidence interval; NA = 3 or less effect sizes in this condition.

[a] Interactive training refers to cognitive activities that require a body-mind interaction such as exergames, square stepping, etc.

[b] Multicomponent training refers to a mixture of cognitive tasks and games, delivered in different modalities such as paper-pencil, verbally, computer-assisted, etc.

* $p < .05$; ** $p < .01$; *** $p < .001$.

Table S6. Results of the continuous and categorical moderator analyses by physical functions.

	Mean difference in ES [95% CI] by moderators in physical outcomes		
	**Fitness**	**Balance**	**Strength**
**Continuous moderators**			
Quality	-0.008 [-0.057, 0.041]	-0.039 [-0.07, -0.008] *	0.015 [-0.066, 0.096]
Year	-0.035 [-0.068, -0.002] *	0.014 [-0.025, 0.053]	-0.051 [-0.155, 0.054]
N	-0.000 [-0.001, 0.000]	-0.001 [-0.002, 0.000]	-0.001 [-0.004, 0.002]
Age mean	-0.016 [-0.06, 0.028]	-0.03 [-0.066, 0.006]	0.038 [-0.041, 0.118]
Age SD	0.029 [-0.057, 0.116]	-0.11 [-0.218, 0.002] *	-0.061 [-0.25, 0.128]
Nº sessions	-0.000 [-0.001, 0.000]	-0.004 [-0.01, 0.002]	-0.012 [-0.025, 0.001]
Training/wks	-0.002 [-0.006, 0.002]	0.001 [-0.006, 0.006]	-0.01 [-0.022, 0.001]
Minutes/week	-0.000 [-0.002, 0.001]	0.000 [-0.001, 0.002]	-0.001 [-0.003, 0.001]
Min. cogn./week	-0.002 [-0.006, 0.002]	0.000 [-0.000, 0.003]	-0.001 [-0.005, 0.002]
Min. phys/week	-0.001 [-0.004, 0.002]	0.001 [-0.001, 0.003]	-0.001 [-0.005, 0.003]
**Combinatory mode**			
Simultaneous	0.184 [-0.021, 0.388]	0.259 [0.153, 0.364] ***	0.334 [0.045, 0.622] *
Sequential	0.151 [-0.095, 0.398]	0.308 [-0.008, 0.623]	0.177 [-0.29, 0.644]
Separate days	0.255 [-0.026, 0.537]	NA	0.147 [-0.429, 0.724]
**Aerobic vs non-aerobic**			
Aerobic	0.257 [0.082, 0.433] **	0.182 [-0.11, 0.475]	0.373 [-0.154, 0.9]
Non-aerobic	0.059 [-0.079, 0.197]	0.272 [0.157, 0.387] ***	0.205 [-0.01, 0.421]
**Cognitive training type**			
Interactive	0.385 [0.113, 0.656] **	0.301 [0.154, 0.449] ***	0.411 [0.086, 0.735] *
Computer	0.04 [-0.187, 0.268]	0.153 [-0.043, 0.343]	0.045 [-0.273, 0.563]
Multicomponent	0.288 [0.102, 0.474] **	0.269 [0.075, 0.464] **	0.4 [-0.096, 0.895]
**Setting**			
Group	0.328 [0.24, 0.453] ***	0.255 [0.12, 0.389] **	0.291 [0.069, 0.512] *
Individual	-0.073 [-0.256, 0.15]	0.242 [0.052, 0.432] *	0.209 [-0.034, 0.452]
Mixed	-0.011 [-0.255, 0.232]	0.394 [-0.04, 0.827]	NA

Note. ES = Hedges' g; CI = confidence interval. NA = not available due to missing effect sizes. * p < .05; ** p < .01; *** p < .001.
